# Automated slope stability assessment using modified Morgenstern-Price method and machine learning integration

**DOI:** 10.1038/s41598-026-38670-w

**Published:** 2026-02-19

**Authors:** Majid Showkat, Sufyan Ghani, Prabhu Paramasivam, Mohamed Yusuf

**Affiliations:** 1 Department of Civil Engineering, Sharda University, Greater Noida, India; 2 Engineer - Tailings (Mine Specialist Team) GCC, WSP, Noida, India; 3 Department of Research and Innovation, Saveetha School of Engineering, SIMATS, Chennai, Tamil Nadu 602105 India; 4 Department of Peace and Development Studies, Njala University, Bo Campus –18, Bo, Sierra Leone

**Keywords:** Slope stability, Morgenstern-Price method, Machine learning, Automated, CatBoost, Factor of safety (*F*_Slope_), Finite slopes, Seismic analysis, Engineering, Mathematics and computing, Natural hazards, Solid Earth sciences

## Abstract

This paper proposes a new hybrid automated framework that combines the Morgenstern-Price Limit Equilibrium Method (MP-LEM) with machine learning to predict the Factor of Safety (*F*_Slope_) for finite slopes under static and seismic loading conditions. The first synthetic data generation involved approximately 100,000 slope conditions that were varied through key geotechnical and seismic parameters: unit weight (γ), slope height (*H*), cohesion (c′), friction angle (ϕ′), slope angle (β), horizontal and vertical seismic coefficients (k_h_ and k_v_), pore pressure ratio (µ), and the interslice force-scaling factor (λ). For model development, a stratified random sample of 20,000 cases was collated to maintain computational feasibility while preserving distributional characteristics. Corresponding *F*_Slope_ values were computed using a simplified force-equilibrium form of the MP-LEM. The novelty of this study lies in the development of a comprehensive automated MP-LEM–ML framework specifically designed for finite slope stability analysis, which integrates a simplified Morgenstern–Price formulation with machine-learning models while explicitly accounting for pore pressure effects, bi-directional seismic loading, and interslice force scaling. The framework also automates the hyperparameter tuning, model evaluation, and optimal model selection within a reproducible Python environment, which enables a practical and deployable system for rapid finite slope stability assessment. Sensitivity analysis found that slope angle (β), slope height (*H*), and cohesion (c′) were the dominant controls on *F*_Slope_. Of nine tested ML algorithms (ANN, RF, DT, KNN, XT, GBoost, AdaBoost, XGBoost, and CatBoost), the CatBoost model had the highest predictive accuracy: R^2^ = 0.999, RMSE = 0.014 on the test set. The MP-LEM implementation was verified further by comparisons to published benchmark results. It returned R^2^ = 0.94, confirming its appropriateness as a reliable deterministic reference. Although the automated framework represents significant computational advantage, it is conditioned by the synthetic dataset based on the 2D and homogeneous soil assumptions, and by the underrepresentation of high-*F*_Slope_ cases. Future work should focus on considering real case histories, probabilistic extensions, and 3D effects for further enhancement of practical applicability. In summary, the hybrid framework proposed herein represents a promising fast, accurate, and scalable decision-support framework for geotechnical slope stability assessment.

**Supplementary Information:**

The online version contains supplementary material available at 10.1038/s41598-026-38670-w.

## Introduction

Slope stability analysis is one of the most important concerns in geotechnical engineering since slope failure is always associated with significant damage to infrastructure and human fatalities. Traditionally, slope stability has been analyzed using analytical and Limit Equilibrium Methods (LEM) in which the Factor of Safety (*F*_Slope_) is calculated against slope instability, based on the balance between the driving and resisting forces^1,2^. Of all these methods, the Morgenstern-Price (MP) method is considered the rigorous one because it satisfies both the force and moment equilibrium and can incorporate any interslice force function^3,4^. Because of its accuracy and flexibility, the MP method has been widely used for natural and engineered slopes under static and seismic loading conditions^5,8^. A summary of representative studies that have implemented the MP method in finite slope analysis is presented in Table 1, highlighting its robustness and wide acceptance in deterministic slope stability evaluation.

**Table 1:** Use of Morgenstern-Price method for *F*_Slope_ in finite slope analysis.

Author(s)	Year	Study focus	Software/Tool	Slope type	Key findings
^2^	2005	Comparative study of slope stability methods	Slope/W (GeoStudio)	Finite slopes in soils	Morgenstern-Price method provides more realistic *F*_Slope_ in complex geometries
^5^	2001	Analysis of reinforced slopes	ReSSA	Reinforced slopes	Morgenstern-Price method accurately models interslice forces
^6^	2008	Influence of pore water pressure in slope stability	Slope/W	Embankments	*F*_Slope_ calculated by MP method sensitive to water pressure assumptions
^9^	2004	Probabilistic analysis with limit equilibrium	Custom FE/LE software	Homogeneous slopes	MP method integrates well with probabilistic frameworks
^7^	2016	Comparison with other methods (Bishop, Janbu)	GeoStudio	Mine slopes	MP method yields conservative and reliable *F*_Slope_

The finite element method (FEM) has been extensively used to simulate the realistic stress–strain response and pore pressure distribution in slope failures beyond the idealizations of the limit equilibrium methods (LEM), as shown in the works of^9,10^. Although LEM is widely applied because of its simplicity and efficiency, stress–strain relationships are explicitly considered in FEM-based methods, although at a much higher computational cost^11^. Moreover, FEM models allow for a comprehensive simulation of coupled mechanical and hydrogeological processes involved in slope failures, although they demand accurate input data, sophisticated constitutive modeling, and high computational power, as emphasized by^12^. More recent works have further combined FEM-based simulations with data-driven models to simulate complex slope deformation processes; however, the high computational cost still hinders their practical applicability for large-scale simulations or real-time simulations, as emphasized in the work of^13^.

In recent times, data-driven approaches, particularly Machine Learning (ML), have emerged as an alternative for rapid and adaptable slope stability prediction. Early applications of ANN (Artificial Neural Network) and SVM (Support Vector Machines) showed notable predictive accuracy relative to conventional regression models^14,15^. Ensemble methods such as RF (Random Forest), GBoost (Gradient Boosting), and XGBoost (eXteme Gradient Boosting) improved generalization and interpretability via feature-importance analyses^16,19^. Hybrid and surrogate models have also been proposed aiming at improving the prediction accuracy by incorporating optimization algorithms into learning strategies^20,22^. Recent studies have also demonstrated that regression models with machine learning capabilities can make direct predictions of the factor of safety in slope stability problems^23^. Representative applications of ML techniques in slope stability prediction are summarized in Table 2, illustrating the evolution from conventional regression to modern ensemble and hybrid algorithms.

**Table 2:** Use of machine learning (ML) in predicting *F*_Slope_ in finite slope analysis.

Author(s)	Year	ML method used	Input parameters	Slope type	Key findings
^14^	2018	Artificial Neural Networks (ANN)	Slope angle, cohesion, friction angle, unit weight	Homogeneous slopes	ANN accurately predicts *F*_Slope_ with > 95% correlation
^15^	2019	Support Vector Machine (SVM)	Soil properties, geometry, water content	Soil slopes	SVM outperforms traditional regression methods
^16^	2020	Decision Tree (DT), Random Forest (RF)	Shear strength, depth, slope height	Natural and cut slopes	RF gives best performance with high feature importance clarity
^17^	2021	XGBoost	Multiple geotechnical inputs	Finite soil slopes	XGBoost showed high prediction accuracy and interpretability
^18^	2022	Hybrid ML (ANN + GA)	Material and design parameters	Road cut slopes	Hybrid model provides optimized, high-accuracy *F*_Slope_ prediction

**Fig. 1:**
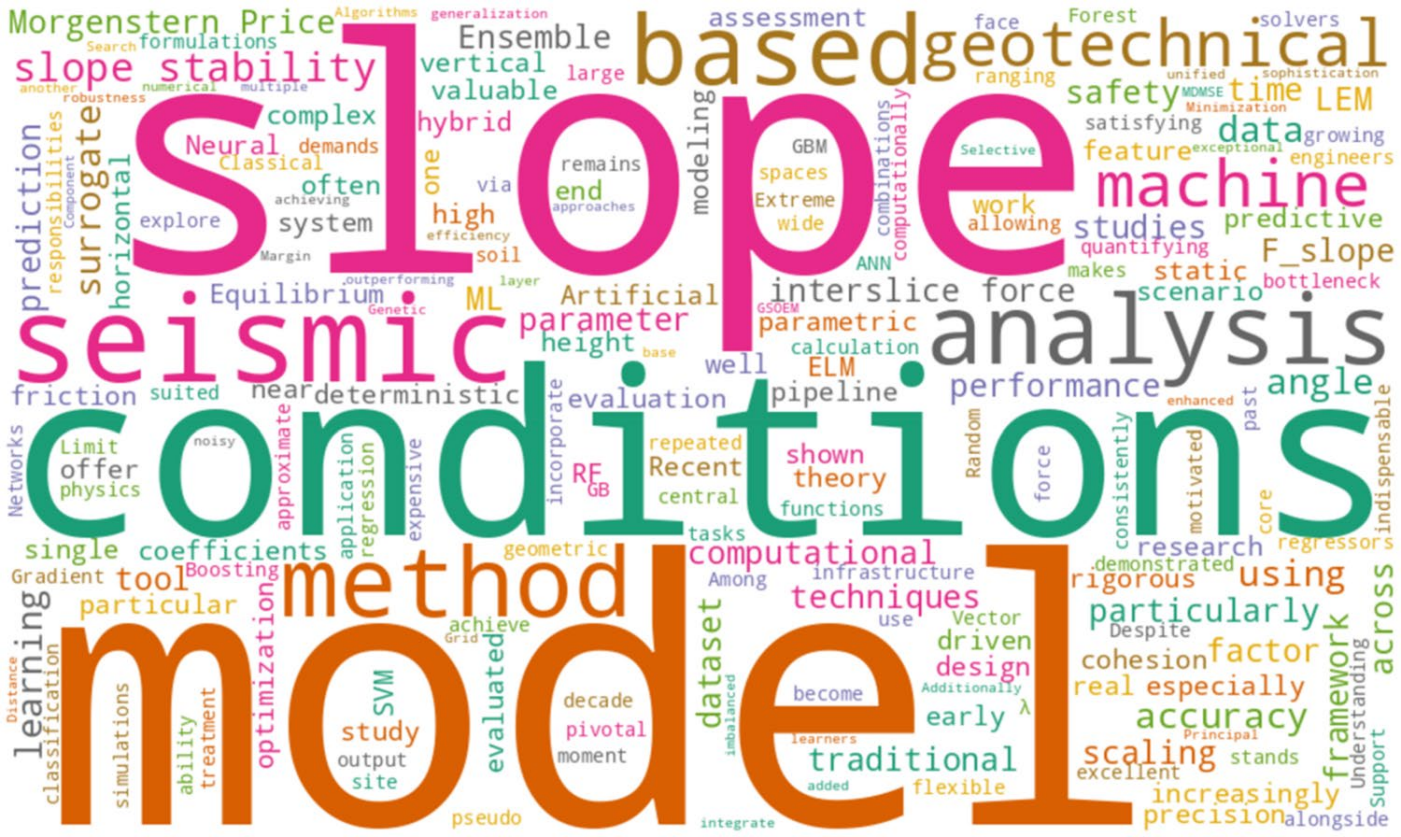
Word cloud of frequently occurring keywords in recent slope stability literature.

Furthermore, the word cloud in Fig. 1 presents a visual overview of the most frequently studied keywords in recent slope stability research, emphasizing the dominance of terms such as *machine learning*, *FEM*, and *slope safety*, which aligns with the current research focus. Despite these developments, ML methods are often plagued by limited physical interpretability and possibly offer poor generalization beyond training data^24^. Recent efforts have overcome these limitations by adopting XAI (Explainable Artificial Intelligence) and physics-informed learning frameworks to combine the interpretability of deterministic theory with the predictive capability of ML. For example^25^, developed a hybrid framework that integrates FEM simulation with ML inference for transparent evaluation of slope stability, while^26^ adopted explainable ML to identify dominant geotechnical parameters influencing the performance of slopes. These works have indicated the emergence of hybridized, physically consistent, and interpretable data-driven models. However, the holistic integration of such deterministic models-the modified MP method-with ML-based automation remains inadequately explored. Most available FEM and ML studies have traditionally focused on either numerical simulation or statistical prediction, with less attention to merging the two paradigms.

As pointed out by^8^ and^20^, deterministic methods, though physically sound, are computationally intensive when handling large parameter spaces, while machine-learning models, although efficient, may lose physical relevance due to limited linkage with the principles of soil mechanics. In response to this research gap, the present study articulates a vision for an automated hybrid framework integrating a modified MP method with state-of-the-art machine-learning algorithms to facilitate accurate and interpretable finite slope stability predictions. Unlike earlier surrogate modeling efforts that primarily focused on infinite slope conditions^27^, the present work addresses finite slopes, which require slice-based equilibrium formulations, non-planar failure mechanisms, and explicit consideration of interslice forces. Correspondingly, this approach involves the generation of a large synthetic slope-scenario dataset using the modified MP method; training and testing multiple machine-learning algorithms, namely ANN, RF, DT (Decision Tree), KNN (k-Nearest Neighbors), XT (Extra Trees), GBoost, AdaBoost (Adaptive Boosting), XGBoost, and CatBoost (Categorical Boosting); and conducting sensitivity analyses to identify dominant influencing parameters. This hybrid Python-based pipeline seeks to couple theoretical rigor with computational efficiency, thereby providing a scalable decision-support tool for slope stability assessment under both static and seismic conditions.

## Data generation and data pre-processing

Data generation and pre-processing for geotechnical slope stability modeling are important processes that determine the accuracy and reliability of predictive models. Data generation typically involves the acquisition of geotechnical parameters of slopes such as soil cohesion, internal friction angle, unit weight, and slope geometry from field measurements and laboratory testing^22^. The raw data are, nonetheless, marred with inconsistencies, redundancies, or missing values, and hence require extensive pre-processing to be of quality and usable. Pre-processing typically involves normalization, missing value handling, and dimensionality reduction^24^. Sound data generation and pre-processing typically form the foundation for effective and reliable slope stability modeling.

### Data preparation and data pre-processing

This study was initiated by generating approximately 100,000 artificial slope-condition instances; the exact number varies slightly due to filtering of non-physical cases and stratified sampling. These instances represent a wide and realistic spectrum of geotechnical and seismic scenarios for finite slope stability analysis. The input parameters included unit weight (γ), slope height (*H*), friction angle (ϕ′), cohesion (c′), slope angle (β), horizontal and vertical seismic coefficients (k_h_ and k_v_), pore pressure ratio (µ), and the interslice force-scaling factor (λ). The factor of safety for all generated cases, denoted as *F*_Slope_, was computed using the simplified Morgenstern–Price method.

**Fig. 2:**
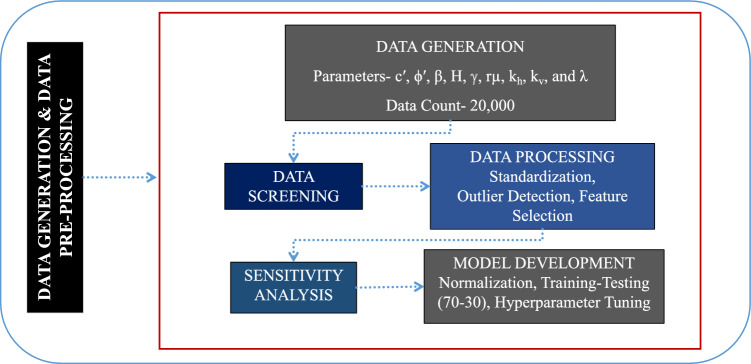
Flowchart illustrating data generation & data pre-processing process.

The complete automated workflow, including data preprocessing, machine-learning training, testing, performance evaluation, and best-model selection, was executed on the full 100,000-case dataset. However, repeated handling of the full dataset for detailed visualization and exploratory analysis resulted in high memory demand. Therefore, a representative subset of 20,000 slope cases was extracted using stratified random sampling to preserve the statistical characteristics of the original dataset, including parameter ranges, variability patterns, and *F*_Slope_ distribution. This subset was used specifically for detailed visualization, sensitivity analysis, and reporting clarity, while the automated modeling process itself remained based on the complete dataset. This strategy ensured computational efficiency in reporting without compromising the integrity of the automated modeling workflow. The overall workflow adopted for synthetic data generation, preprocessing, and dataset preparation is illustrated in Fig. 2.

**Fig. 3:**
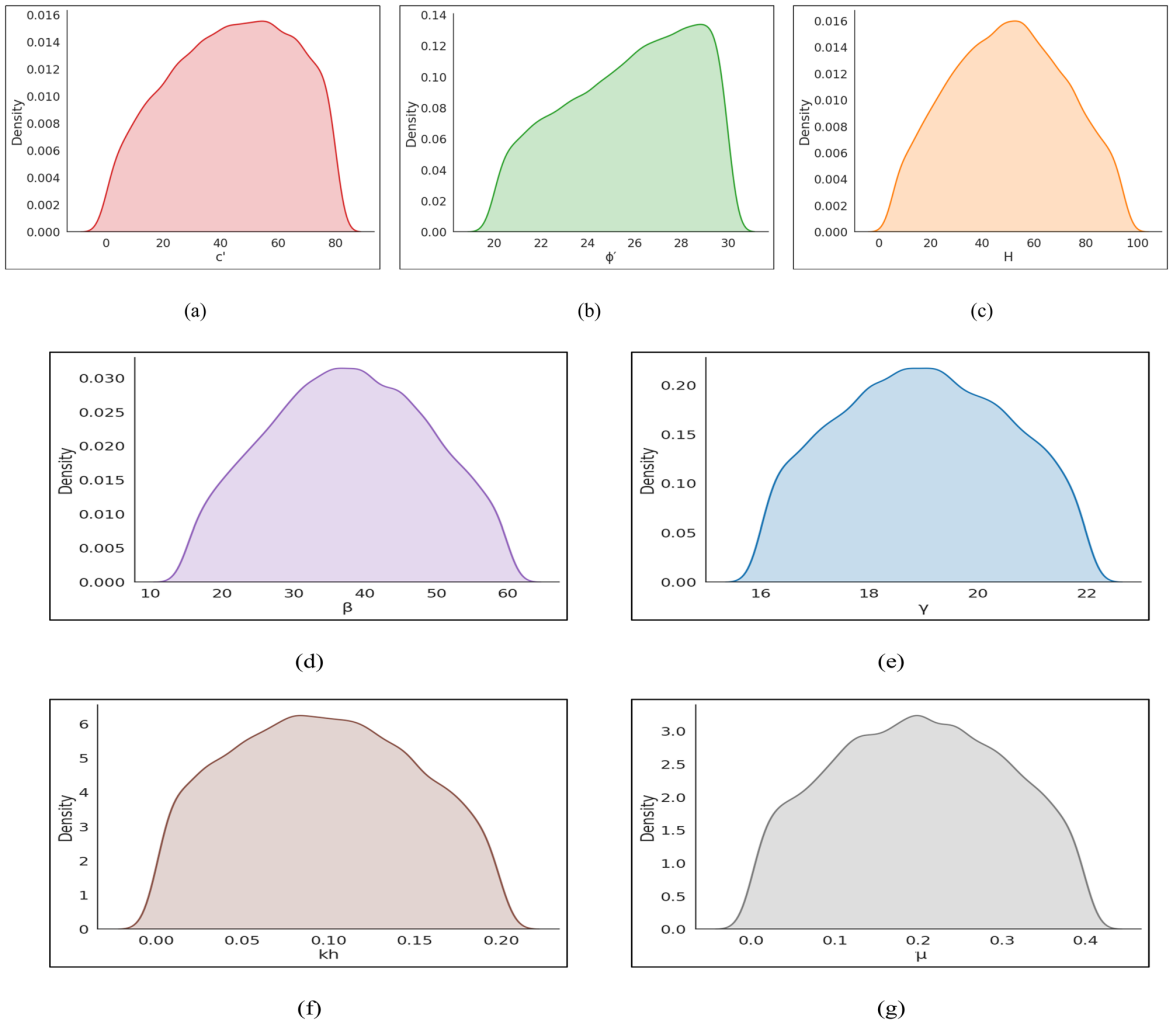

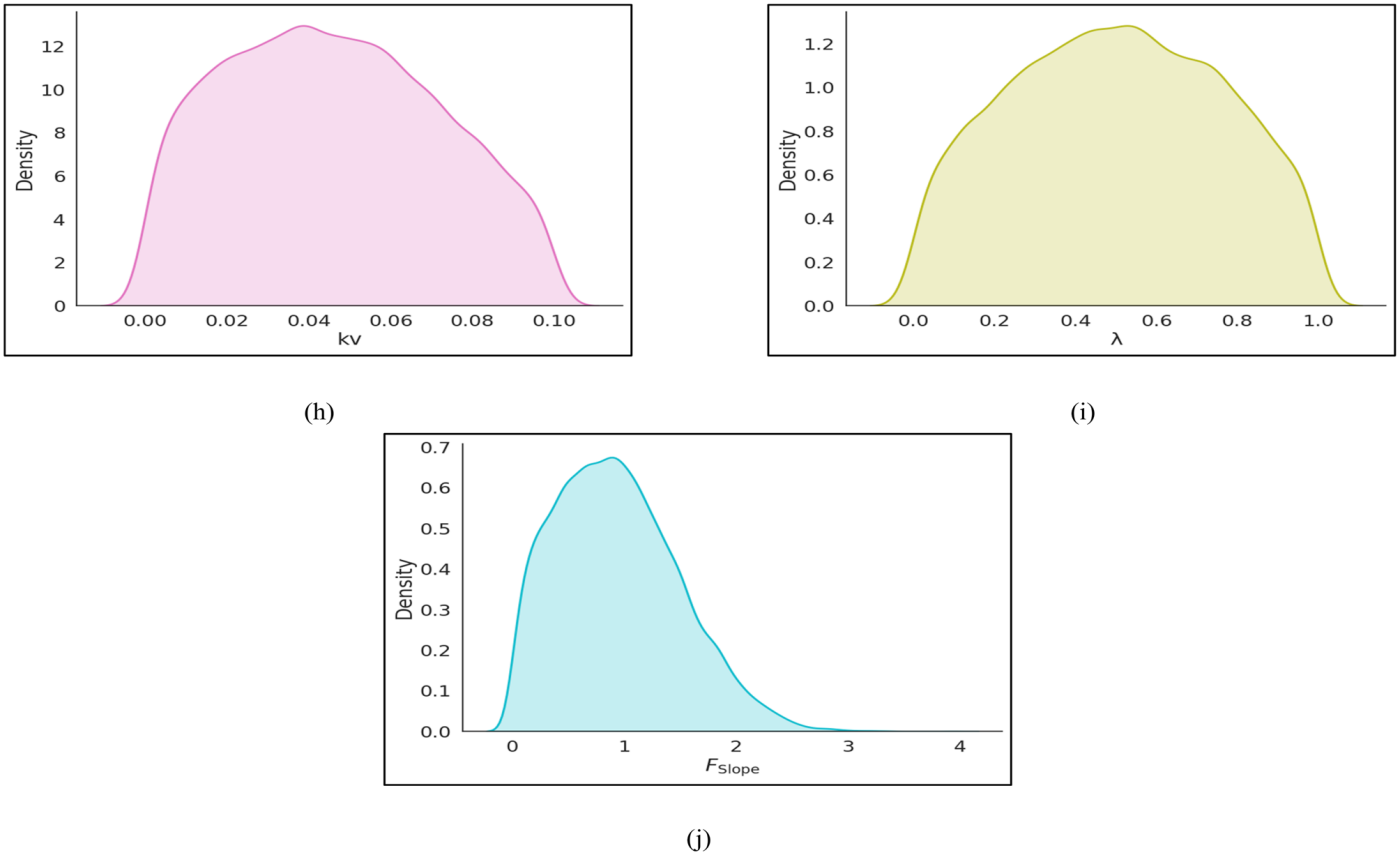
Density plots (**a**) c′ (**b**) ϕ′ (**c**) *H* (**d**) β (**e**) γ (**f**) k_h_ (**g**) µ (**h**) k_v_ (**i**) λ (**j**) *F*_Slope_.

The selection of parameter ranges and preprocessing strategies was guided by the objective of capturing realistic finite-slope conditions commonly encountered in geotechnical engineering practice, while ensuring statistical robustness for machine-learning modeling. The ranges for all geotechnical, geometric, hydraulic, and seismic parameters were estimated from published literature and from limits generally adopted in engineering practice, and their engineering rationale together with key supporting references are summarized in Table 3. A statistical summary of the dataset is provided in Table 4. Density plots were drawn for all input variables (γ, H, ϕ′, c′, β, k_h_, k_v_, µ, λ) and for *F*_Slope_ to explore the underlying structure of the dataset (Fig. 3). Most parameters exhibited unimodal or near-normal distributions with moderate skewness, which is consistent with natural geotechnical variability observed in field conditions.

**Table 3:** Geotechnical parameter ranges used for synthetic slope dataset generation and their engineering rationale.

Parameter	Range	Engineering rationale	Key references
γ (kN/m^3^)	16–22	Typical range for natural soils and compacted fills	^2,16^
c′ (kPa)	0–80	Covers cohesionless soils to stiff clays	^6,7^
ϕ′ (°)	20–30	Representative of sandy and silty soils	^2^
H (m)	5–95	Highway cuts, embankments, mine slopes	^5,7^
β (°)	15–60	Natural to steep engineered slopes	^8^
µ	0–0.4	Dry to near-saturated field conditions	^6^
k_h_	0–0.2	Pseudo-static seismic loading	^28^
k_v_	0–0.1	Vertical seismic effects	^28^
λ	0–1	Full admissible interslice force range	^3,4^

**Table 4:** Summary of statistical values of the dataset.

Category Symbols	Input									Output
	γ	*H*	ϕ′	c′	β	k_h_	k_v_	µ	λ	(*F*_Slope_)
Counts	20,000	20,000	20,000	20,000	20,000	20,000	20,000	20,000	20,000	20,000
Max	22	95	30	80	60	0.2	0.1	0.4	1	6.47
Min	16	5	20	0	15	0	0	0	0	0.001
Mean	18.99	49.86	25.05	39.85	37.56	0.09	0.04	0.2	0.49	0.78
Median	19	45	25	35	40	0.1	0.05	0.2	0.5	0.59
Std. Dev	2.23	28.64	3.54	23.05	14.32	0.08	0.04	0.16	0.4	0.66

Outliers were treated using engineering-realistic capping rules, such as cohesion capped at 80 kPa and friction angle capped at 30°, while missing values were imputed or excluded when non-inferable. All numerical variables were normalized using the min–max method to ensure consistent scaling for machine-learning models, particularly those sensitive to feature magnitude. The cleaned 20,000 dataset was then split into training (T_RG_) and testing (T_SG_) subsets using stratified sampling based on *F*_Slope_ to ensure proportional representation of unstable, marginally stable, and stable slope conditions in both subsets. All preprocessing steps were performed in Python using NumPy, Pandas, and Scikit-learn libraries, with a fixed random seed to ensure reproducibility of sampling and model partitioning. Correlation checks were conducted to verify that no significant multicollinearity existed among input features. These preprocessing measures ensured that the dataset remained physically realistic, statistically representative, and suitable for robust training and evaluation of machine-learning models.

### Sensitivity analysis

The sensitivity analysis was conducted using a global, model-based approach derived from trained tree-based ensemble machine-learning models, rather than through direct perturbation of the Morgenstern–Price equations or variance-based sensitivity methods. Parameter importance scores were obtained from normalized feature-importance measures produced by ensemble learners, primarily from the Random Forest and Gradient Boosting family models. These importance values quantify the relative reduction in prediction error contributed by each input variable across the entire parameter space and therefore represent a global sensitivity measure, rather than localized variations around a single reference condition. To enhance robustness, importance trends were cross-checked across multiple ensemble learners and were found to be consistent.

**Fig. 4:**
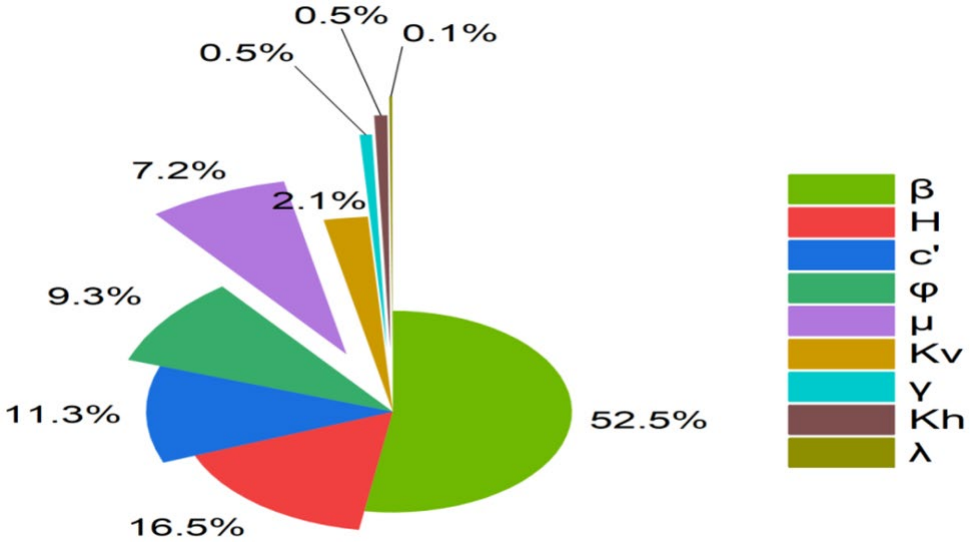
Pie chart illustrating the percentage contribution of each parameter to *F*_Slope_.

This sensitivity framework assumes independent variation of input parameters within their prescribed ranges, which is consistent with the adopted synthetic data generation strategy. The analysis evaluates the relative influence of key geotechnical, geometric, hydraulic, and seismic parameters, including slope height (*H*), slope angle (β), cohesion (c′), friction angle (ϕ′), pore pressure ratio (µ), horizontal and vertical seismic coefficients (k_h_ and k_v_), unit weight (γ), and the interslice force-scaling factor (λ) on the factor of safety, *F*_Slope_. The percentage contribution of each parameter is illustrated in Fig. 4, and the corresponding importance scores are summarized in Table 5.

**Table 5:** Table summarizing sensitivity of parameters towards *F*_Slope_.

Parameter	Importance score
β	0.51
*H*	0.16
cʹ	0.11
ϕʹ	0.09
µ	0.07
k_v_	0.02
γ	0.005
k_h_	0.005
λ	0.001

As shown in Table 5, β is the most influential parameter, with an importance score of 0.51, indicating that slope steepness exerts dominant control on stability by significantly increasing driving forces along the failure surface. H follows with an importance score of 0.16, reflecting the increase in shear stress associated with larger soil masses. c′ and friction angle ϕ′, with importance scores of 0.11 and 0.09, respectively, represent key components of soil shear strength and play a critical role in slope stability, particularly in fine-grained or weak soil conditions. The pore pressure ratio µ exhibits a moderate influence 0.07, highlighting the reduction in effective stress under saturated or rainfall-induced conditions.

Seismic effects, represented by the vertical (k_v_ = 0.02) and horizontal (k_h_ = 0.005) seismic coefficients, show comparatively lower sensitivity under typical conditions but remain important in seismically active regions or under strong ground motion. γ and the λ exhibit minimal sensitivity within the investigated parameter ranges, indicating limited direct influence on *F*_Slope_ under normal conditions, although they remain relevant for detailed equilibrium calculations and specific design scenarios.

From a design and engineering perspective, these results provide practical guidance for slope stability assessment and mitigation planning. Parameters with high sensitivity, particularly β, H, and c′ should be prioritized during site investigation, monitoring, and design optimization, as relatively small variations can lead to significant changes in *F*_Slope_. Secondary parameters such as ϕ′ and µ become critical under adverse hydraulic or material conditions and should therefore be carefully evaluated in rainfall-prone or saturated slopes. Parameters with lower sensitivity may have limited influence under routine conditions but remain relevant for detailed design checks and seismic assessments. Overall, the sensitivity trends align well with established geotechnical principles, reinforcing the dominant role of slope geometry and shear strength in practical slope design.

While advanced interpretability techniques such as SHAP values or partial dependence plots were not explicitly applied in this study, model interpretability is effectively addressed through the global sensitivity analysis based on ensemble-derived feature importance. The dominance of β, H, and c′ identified by this analysis is physically consistent with classical slope stability theory, thereby providing meaningful insight into machine-learning behavior. The incorporation of local, instance-level interpretability tools is identified as an important extension for future work to further enhance transparency and engineering insight.

### Model development

The data were pre-processed using min–max normalization to ensure numerical stability and consistent input scaling for machine-learning models sensitive to feature magnitude, such as ANN and distance-based learners. While tree-based ensemble models (RF, DT, XT, boosting-based methods) are largely insensitive to feature scaling, a unified preprocessing strategy was adopted to maintain consistency across models and facilitate comparative evaluation. To prevent the impact of varying units and magnitudes during model training, the transformation normalized all input features to between 0 and 1. For models sensitive to input scale, such as ANN and distance-based learners, normalization is particularly important, whereas tree-based models benefit primarily from consistent preprocessing rather than scale dependence. Skewness in the feature distributions was prevented through the transformation of the dataset, giving each parameter an equal contribution to the prediction of the *F*_Slope_ of finite slope stability.

The data were partitioned into training (T_RG_) and testing (T_SG_) subsets by stratified sampling in manner such that proportionate splitting of *F*_Slope_ values and other geotechnical parameters was maintained. The 70% cumulative data proportion was used as the T_RG_ subset for machine learning model training while the rest, 30%, was left as the T_SG_ dataset for evaluation of generalization performance. Proportionate splitting maintained during data division ensured good evaluation while bias during prediction analysis was prevented to the best. Ensemble learning models and traditional ML models have been utilized to predict *F*_Slope_ for finite slope stability.

All the models were extensively hyperparameter tuned to obtain maximum predictability. Cross-validation and grid search methods were applied to tune key hyperparameters in order to avoid overfitting and optimize computational efficiency. The hyperparameters tuned for all the models are listed in Table 6.

**Table 6:** Hyperparameter settings of the models’ employed.

Model	Tuned hyperparameters
ANN (artificial neural network)	Architecture: 3 hidden layers; Neurons: [128, 64, 32]; Activation Function: ReLU; Optimizer Used: Adam; Learning Rate: 0.001; Epochs: 100; Batch Size: 32
RF (random forest)	Total Trees: 500; Tree Depth: 10 (max); Minimum Split Samples: 2; Leaf Sample Minimum: 1; Feature Selection Strategy: √ (sqrt); Bootstrapping: Enabled
GBoost (gradient boosting)	Estimator Count: 500; Learning Rate Applied: 0.05; Maximum Depth: 5; Min. Samples for Split: 2; Minimum Leaf Samples: 1
AdaBoost	Number of Weak Learners: 500; Learning Rate: 0.05; Base Model: Decision Tree Classifier
CatBoost	Iterations: 1000; Learning Rate: 0.03; Tree Depth: 6; Regularization (L2 Leaf): 3; Bagging Temp.: 1.0; Subsampling Ratio: 0.8
XGBoost	Boosting Rounds: 500; Learning Rate Set: 0.05; Maximum Tree Depth: 6; Gamma Value: 0; Child Weight (Min): 1; Row Subsample: 80%
DT (decision tree)	Max Tree Depth: Not Limited; Minimum Samples to Split: 2; Minimum Leaf Samples: 1
XT (extra trees)	Trees in Ensemble: 500; Maximum Depth: Not Constrained; Min. Split Samples: 2; Min. Leaf Samples: 1; Feature Consideration: √; Bootstrapping: Disabled
KNN (K-nearest neighbors)	K Value (Neighbors): 5; Weight Function: Uniform; Distance Metric: Minkowski; Power Parameter (p): 2

The machine learning model simulations were performed in Python (v3.11), predominantly using open-source libraries such as NumPy, Pandas, Scikit-learn, and Matplotlib. The complete dataset generated from the modified Morgenstern–Price analysis was randomly divided into 70% for training and 30% for testing, ensuring that both subsets maintained similar statistical distributions of geotechnical and seismic parameters. Model training was conducted under a supervised regression framework, where each algorithm iteratively optimized its parameters to minimize prediction error between the predicted and reference *F*_Slope_ values.

**Fig. 5:**
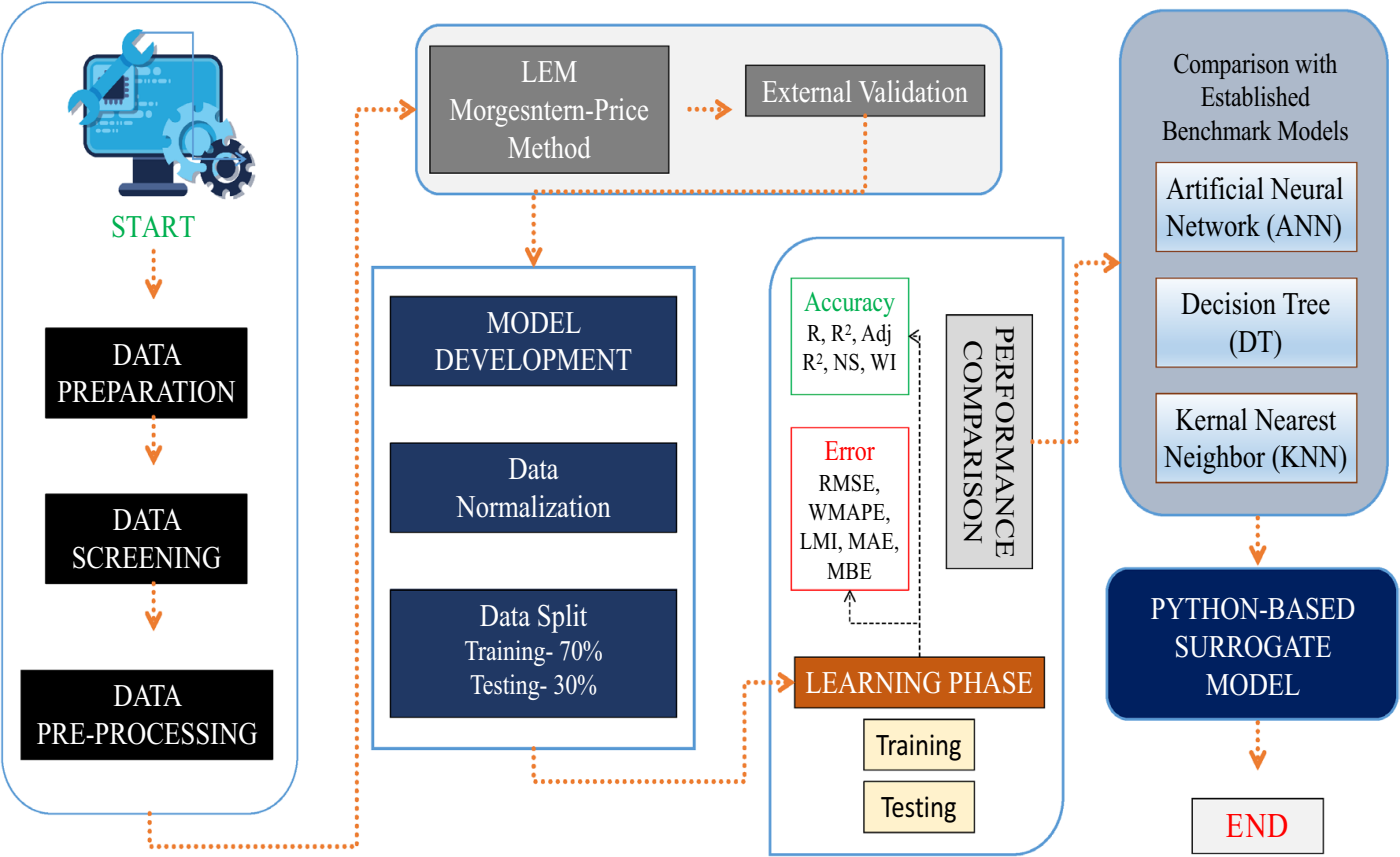
Flowchart illustrating the methodology workflow.

To ensure reproducibility and robustness, the simulations were repeated across multiple random states, and the results were averaged. During the training phase, convergence was monitored by tracking training and testing errors to verify stability and prevent overfitting. The best-performing configuration for each model was selected based on minimum testing RMSE and maximum R^2^ values. This simulation protocol ensured reliable, reproducible, and computationally efficient evaluation of all machine learning models within the hybrid modified MP–ML framework. A schematic representation of the overall model training and evaluation process is presented in Fig. 5, illustrating the data flow from deterministic computation to ML simulation. To ensure training stability and optimal learning behavior, the convergence performance of each model was monitored by tracking loss reduction over successive epochs or iterations. Detailed analysis is presented in Section “ML results’s” convergence analysis.

## Methodology

This research places finite slope stability analysis in a holistic context, unifying traditional geotechnical methods with modern ML methods. Through the integration of the Morgenstern-Price LEM with modern ML methods, this context not only provides improved prognostic performance and accuracy but also circumvents some of the drawbacks of traditional slope stability analysis methods. Perhaps foremost, it offers a well-defined and pragmatic context for actual use in geotechnical engineering.

The approach, as shown in Fig. 5, starts with the computation and identification of nine geotechnical parameters, i.e., c′, ϕ′, γ, H, β, k_h_, k_v_, µ, and λ. These parameters are the central pillar for both conventional and ML-based methods, providing a complete analysis of slope stability under different geological and environmental conditions.

This research puts finite slope stability analysis within an integrated framework that brings together traditional geotechnical engineering practices and state-of-the-art machine learning models. With the coupling of the application of the Morgenstern-Price model with state-of-the-art machine learning models, this framework seeks to improve predictive accuracy as well as computational efficiency and circumvent some of the limitations of traditional practices. Above all else, this method offers a systematic and practical framework for routine slope stability analysis and daily decision-making.

### Simplified Morgenstern-Price method

The Morgenstern-Price slope stability analysis is based on partitioning of potential sliding mass into a series of vertical slices and imposition of equilibrium conditions on each slice. For this research, a force-equilibrium-based simplification is derived from the original work of^3^, as practiced by^29^. The derivation below leads in a step-by-step manner to the final expression of *F*_Slope_ used in this research.

**Fig. 6:**
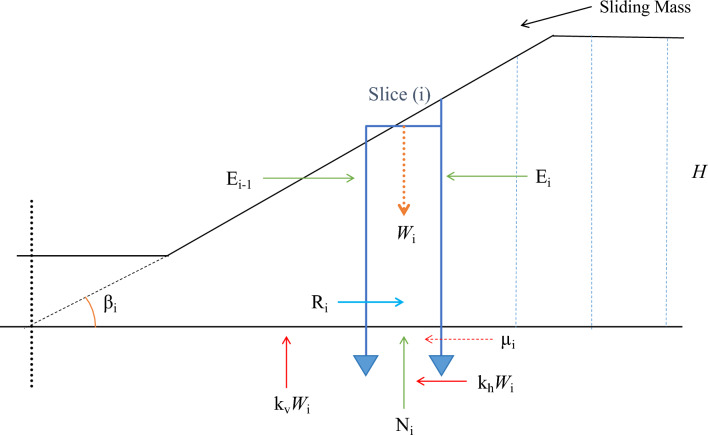
Illustrating finite slope with a sliding mass and slice-based force system.

Consider a typical vertical slice within the potential failure mass (Fig. 6).

where,

*W*_i_ is the weight of the slice, acting vertically downward; E_i−1_ and E_i_ are the interslice forces (normal and shear components) on the left and right sides; R_i_ denotes shear resistance mobilized at the base of the slice; N_i_ is the normal force at the base of the slice; µi is pore water pressure acting at the base; k_h_*W*_i_ and k_v_*W*_i_ are the horizontal and vertical seismic forces due to earthquake shaking respectively; and the slice base is inclined at an angle β to the horizontal.

Force equilibrium in the X-direction (parallel to slope):

1 $${W}_{i}sin{\beta }_{i}+{k}_{h}{W}_{i}cos{\beta }_{i}+({E}_{i}-{E}_{i-1})={R}_{i}$$

where; *W*_i_sin*β*_i_ is the component of weight driving the motion; k_h_*W*_i_cos*β*_i_ is the horizontal seismic force; E_i_ − E_i-1_ represents the difference in horizontal interslice forces; R_i_ is the resisting shear force along the base.

Force equilibrium in the Y-direction (normal to slope):

2 $${W}_{i}cos{\beta }_{i}+{k}_{v}{W}_{i}-\left\{{N}_{i}+({E}_{i-1}+{E}_{i})sin{\alpha }_{i}\right\}=0$$

where, α_i_ is the assumed inclination of interslice normal forces.

In a simplified force equilibrium formulation, the contribution of E_i_ terms to vertical equilibrium can be neglected by appropriate selection of the interslice function or scaling.

According to the Mohr–Coulomb failure criterion, the available shear strength S_i_ at the base of slice *i* is:

3 $$S_{i} = c_{i}^{\prime } + \left( {\sigma_{i}^{\prime } } \right)tan \phi_{i}^{\prime }$$

where; c_i_′ is the effective cohesion; σ_i_′effective normal stress on the base; ϕ_i_′ is effective angle of internal friction.

The effective normal stress σ_i_′ is:

4 $${\sigma^{\prime}}_{i}=\frac{{N}_{i}}{{L}_{i}}-{\mu }_{i}$$

with; N_i_ is total normal force; L_i_ is length of the base of the slice; µ_i_ is pore water pressure at the base.

However, N_i_ can be approximated by:

5 $${N}_{i}\approx {W}_{i}cos{\beta }_{i}$$

under small interslice shear assumptions (neglecting interslice shear contribution for simplification). Thus,

6 $${\sigma^{\prime}}_{i}=\frac{{W}_{i}cos{\beta }_{i}}{{L}_{i}}-{\mu }_{i}$$

Substituting into the Mohr–Coulomb equation:

7 $${S}_{i}={c^{\prime}}_{i}+\left(\frac{{W}_{i}cos{\beta }_{i}}{{L}_{i}}-{\mu }_{i}\right)tan{\phi^{\prime}}_{i}$$

The total resisting force on the base of the slice, considering the interslice force scaling factor λ_i_, becomes:

8 $${Resisting\;Force}_{i}={\lambda }_{i}\times {S}_{i}\times {L}_{i}$$

Substituting the expression for S_i_:

9 $${Resisting\;Force}_{i}={\lambda }_{i} \left\{{c^{\prime}}_{i}{L}_{i}+\left(\frac{{W}_{i}cos{\beta }_{i}}{{L}_{i}}-{\mu }_{i}\right)tan{\phi^{\prime}}_{i}\times {L}_{i}\right\}$$

Simplifying:

10 $${Resisting\;Force}_{i}={\lambda }_{i} \left\{{c^{\prime}}_{i}{L}_{i}+\left({W}_{i}cos{\beta }_{i}-{\mu }_{i}{L}_{i}\right)tan{\phi^{\prime}}_{i}\right\}$$

The total driving force acting along the base direction includes the downslope component of the weight *W*_i_sin*β*_i_; seismic inertial force k_h_*W*_i_cos*β*_i_; vertical seismic correction k_v_*W*_i_. Thus,

11 $${\mathrm{Driving}}\;{\mathrm{Force}}_{{\mathrm{i}}} = {\mathrm{W}}_{{\mathrm{i}}} (1 - {\mathrm{k}}_{{\mathrm{v}}} )\;\sin \beta_{i} + {\mathrm{k}}_{{\mathrm{h}}} {\mathrm{W}}_{i} \cos \beta_{i}$$

Summing the resisting forces and the driving forces over all *n* slices:

12 $${F}_{Slope}=\frac{{\sum }_{i=1}^{n}{Resisting\;Force}_{i}}{{\sum }_{i=1}^{n}{Driving\;Force}_{i}}$$

Substituting the expanded expressions:

13 $${F}_{Slope}=\frac{\sum_{i=1}^{n}{\lambda }_{i}\left\{{c^{\prime}}_{i}{L}_{i}+\left(\frac{{W}_{i}cos{\beta }_{i}}{{L}_{i}}-{u}_{i}\right)tan{\phi^{\prime}}_{i}\right\}}{{\sum }_{i=1}^{n}\left\{{W}_{i}\left(sin{\beta }_{i}+{k}_{h}cos{\beta }_{i}\right)+{k}_{v}{W}_{i}\right\}}$$

In this case, the interslice normal forces are indirectly represented by the scaling factor λ_i_, and the slope height *H* influences the calculations of weight for slices but does not directly appear in the governing equations. Seismic forces, in the form of horizontal and vertical seismic coefficients (k_h_ and k_v_), and pore water pressure (µ_i_), are directly represented in the analysis. The approach is generally a reasonable balance between theoretical and practical simplicity and is therefore particularly suitable to automatic data generation for machine learning purposes.

The simplified Morgenstern–Price formulation adopted in this study is based on a force-equilibrium framework and is intended to balance theoretical rigor with computational efficiency. Several assumptions are inherent in this simplification and are explicitly acknowledged here to aid interpretation, particularly for readers less familiar with limit equilibrium methods.

First, the formulation assumes two-dimensional slope geometry with a predefined slip surface and homogeneous soil properties within each slice. Three-dimensional effects, spatial variability of material properties, and progressive failure mechanisms are not considered. Second, interslice shear forces are neglected, and the influence of interslice normal forces is incorporated indirectly through a scalar interslice force scaling factor (λ). While this approach is consistent with commonly adopted simplified MP implementations^29^, it does not capture the full flexibility of arbitrary interslice force functions available in the original Morgenstern–Price method^3^.

Third, seismic effects are represented using a pseudo-static approach, where horizontal and vertical seismic coefficients are treated as constant body forces. This approximation neglects dynamic amplification, phase effects, and time-dependent soil response, and is therefore most appropriate for preliminary seismic stability assessments rather than detailed dynamic analyses. Similarly, pore water pressure is represented using a pore pressure ratio, which assumes steady-state or simplified hydraulic conditions and does not explicitly model transient seepage or unsaturated flow behavior.

Despite these limitations, the simplified formulation retains the essential physics governing slope stability and has been shown to produce reliable *F*_Slope_ estimates for a wide range of practical conditions. Its reduced computational complexity makes it particularly suitable for large-scale parametric studies and automated dataset generation, as required for machine-learning-based surrogate modeling. The approach should therefore be interpreted as a screening- and decision-support tool, rather than a replacement for detailed finite element or fully rigorous limit equilibrium analyses in critical design scenarios.

### Machine learning modeling

ML methodologies were utilized to create forecasting models for the estimation of the *F*_Slope_ of geotechnical slopes based on more than one input parameter. Pre-processing of data involving min–max normalization and outliers treatment was followed by the division of the dataset into T_RG_ and T_SG_ subsets to facilitate stable model assessment. Various ML algorithms, such as ANN, DT, KNN (benchmark models), RF and XT (bagging models) and AdaBoost, GBoost, CatBoost, and XGBoost (boosting models), were tried. The models were trained to identify the correlation between geotechnical parameters such as unit weight, cohesion, internal friction angle, slope geometry, seismic loading and resultant *F*_Slope_ values. The machine learning models utilized and their architectures are described in detail in^27^.

To assess predictive capability and generalizability of all models, a complete set of evaluation metrics was utilized. These included both error and statistical metrics: Pearson correlation coefficient (r), coefficient of determination (R^2^), adjusted R^2^, root mean square error (RMSE), weighted mean absolute percentage error (WMAPE), mean absolute error (MAE), mean bias error (MBE), Nash–Sutcliffe efficiency (NS), Legates and McCabe Index (LMI), and Willmott’s Index of Agreement (WI). Multi-metric approach to model performance measurement allowed performance to be assessed from a variety of different perspectives i.e., accuracy, bias, consistency, and efficiency, providing a general overview of each algorithms’ performance.

### Python-based automated framework

To facilitate effective streamlining of the slope stability prediction procedure, we developed an automatic Python-based framework. The framework carries out the entire modeling pipeline, including generation of synthetic data and normalization, application of the ML model, performance evaluation, and model selection. The framework employs Python’s scientific computing platform; predominantly libraries such as NumPy, Pandas, Scikit-learn, and Matplotlib to manage data effectively, execute T_RG_, T_SG_, and visualization.

The automated process was designed to mimic traditional analysis methods but much quicker and more versatile. Once the models are trained, the system evaluates all the candidates against the set performance standards and selects the best-performing model based on a composite score in an automatic manner. It delivers consistent and unbiased selection criteria without the hassle of bulk manual comparisons. The automated process not only accelerates the analysis but also offers a reusable and scalable method for future slope stability analysis for various geotechnical conditions.

## Results and discussion

This section explains the outcome of the finite slope analysis, in which three important steps have been accomplished: the calculation of the *F*_Slope_ by Morgenstern-Price method and its validation with a published work, ML analysis and model performance, and the creation of a Python-based automated model. The analysis starts with LEM, which calculates the *F*_Slope_ by Morgenstern-Price LEM method and verification. It is then followed by the use of ML models to predict the *F*_Slope_ using various geotechnical parameters and geometrical parameters, where their performance is assessed on the basis of various performance measures. Lastly, to automate and ease the process, a Python-based automated model is created, where both Morgenstern-Price calculations and ML predictions have been merged into an executable script. It offers improved computational speed and eliminates the use of manual procedure, hence situating slope stability evaluation within easier understanding and more easily within practical implementation in geotechnical engineering.

### LEM analysis

**Fig. 7:**
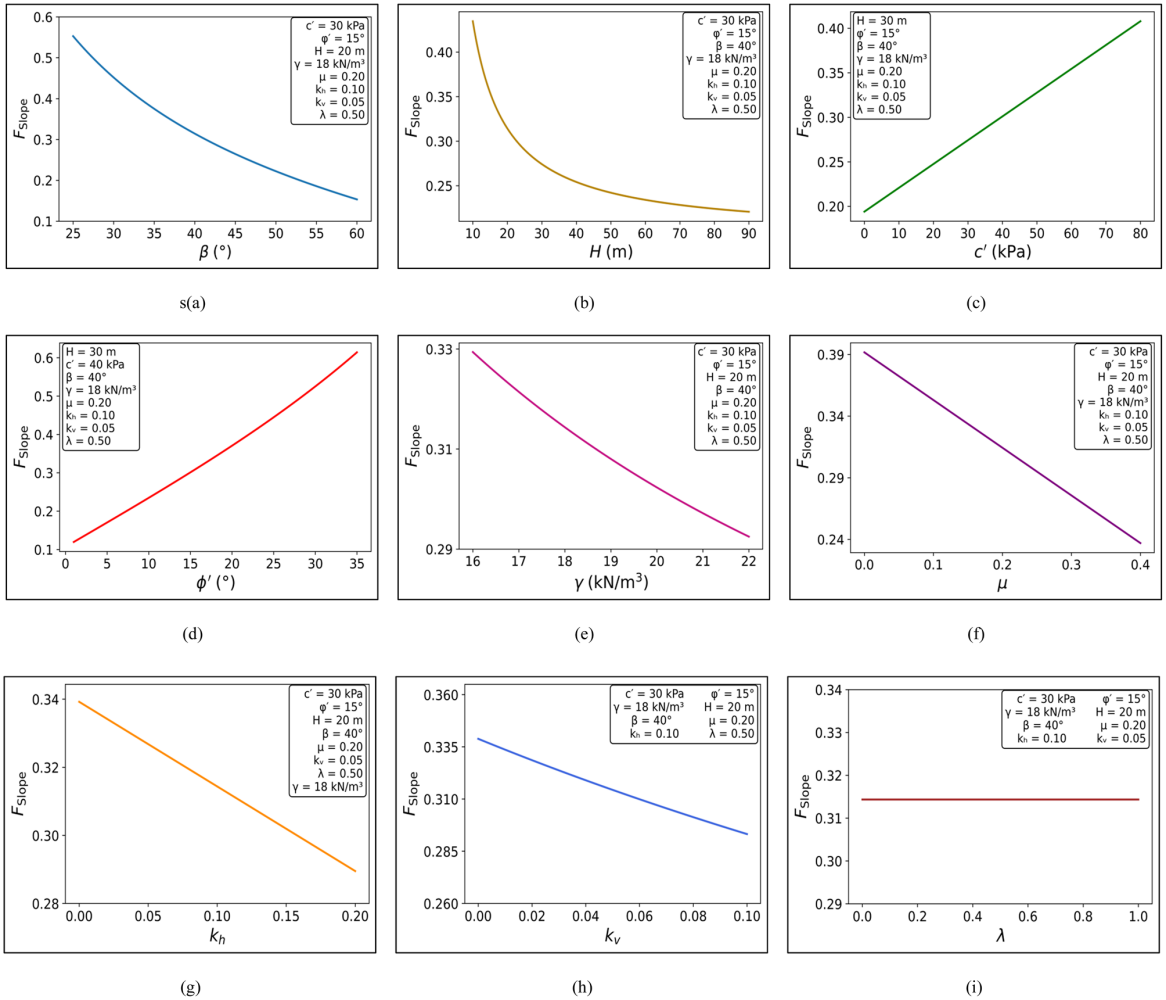
Line plot showing effect of each parameter on *F*_Slope_ (**a**) slope angle (**b**) slope height (**c**) cohesion (**d**) friction angle (**e**) unit weight (**f**) pore water pressure (**g**) horizontal seismic coefficient (**h**) vertical seismic coefficient (**i**) interslice force scaling factor.

In this present analysis, *F*_Slope_ was experimented with varying the slope parameters while maintaining constant other parameters. The following cases were experimented with:

β was varied from 25° to 60° with c′ constant at 30 kPa, ϕ′ constant at 15°, H constant at 20 m, γ constant at 18 kN/m^3^, µ constant at 0.2, kv constant at 0.05, k_h_ constant at 0.1, and λ constant at 0.5. The trend indicated the *F*_Slope_ to decrease with an increase in β as can be seen from Fig. 7a. Again, H was varied from 5 to 95 m for the constant c′ values of 30 kPa with other parameters constant. The *F*_Slope_ was found to decrease with an increase in H, hence providing the effect of slope geometry. c′ values were varied from 5 to 90 kPa while *H* was constant at 30 m. This provided a direct proportion for c′ and *F*_Slope_, meaning that larger values of c′ enhance the slope stability. The value of ϕ′ was varied from 5° to 35° with constant c′ of 40 kPa and constant H of 30 m. It was observed that *F*_Slope_ increased with, providing the effect of the shear strength parameters on the stability of the slope.

When γ was changed from 16 to 22 kN/m^3^ while all other parameters remained constant. The result indicated γ increase resulted in *F*_Slope_ decrease, which indicates the effect of self-weight on stability. The µ was changed from 0 to 0.4. The result indicated that as µ increased, *F*_Slope_ decreased, which indicates the effect of destabilizing water pressure. The k_h_ was changed from 0 to 0.2, but the result indicated little difference in *F*_Slope_, which indicates little effect of k_h_ under conditions studied. The k_v_ was changed from 0 to 0.1, and as k_v_ increased, *F*_Slope_ decreased, which indicates the effect of destabilizing vertical seismic forces. The λ was varied from 0 to 1, and the results confirmed that *F*_Slope_ remained unchanged. This indicates that interslice forces do not influence slope stability under the adopted force-equilibrium-based Morgenstern–Price formulation.

The LEM solution using the Morgenstern–Price algorithm demonstrates that *F*_Slope_ is sensitive to several geometric and geotechnical parameters (Fig. 7). Slope stability increases with increasing cohesion and friction angle, while it decreases with increasing *H* and β. *F*_Slope_ also decreases as unit weight, pore water pressure ratio, and vertical seismic coefficient increase. In contrast, *F*_Slope_ was found to be weakly sensitive to variations in the k_h_ for the investigated parameter range. Overall, the results provide valuable insights for slope stability assessment and practical slope design in geotechnical engineering.

The outcomes of the Morgenstern-Price method were contrasted with conventional benchmarks in terms of confirming the reliability and accuracy of the *F*_Slope_ determined for finite slopes. The Morgenstern-Price method is a traditional LEM in the determination of interslice forces and is founded upon both force and moment equilibrium and an arbitrary function. This makes the stability analysis of slopes the conventional choice of geotechnical research and more accurate.

**Fig. 8:**
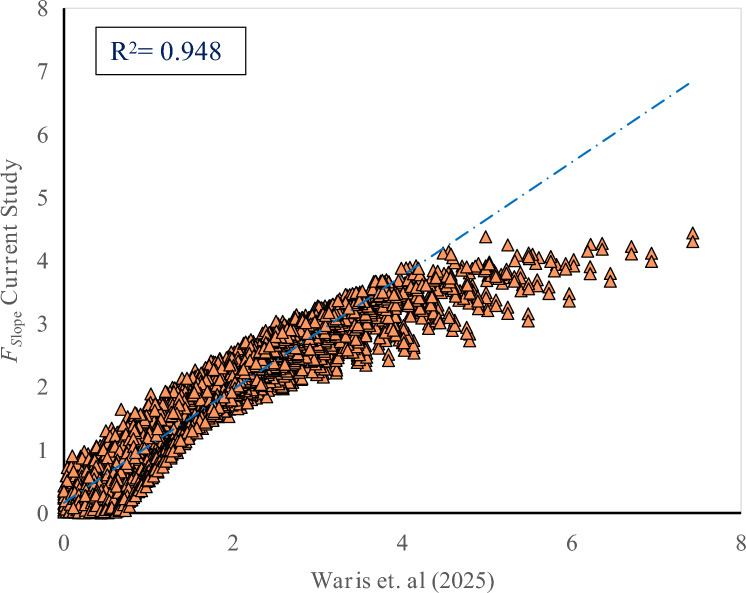
*F*_Slope_ comparison- current study vs Waris et al. (2025).

External validation of the proposed *F*_Slope_ computation was carried out by comparing values obtained using the modified Morgenstern–Price method with reference values reported by^28^. The Waris et al. dataset, which was not involved in the model development process, served as an independent benchmark to verify the accuracy and generalization capability of the proposed approach. The aim was to prove the accuracy and correlation of the computed *F*_Slope_ values through statistical validation methods. To measure the accuracy of the computed *F*_Slope_ values, the coefficient of determination (R^2^) was employed. The R^2^ that gauges the goodness of fit between the predicted and observed results was computed to be 94% (Fig. 8). This high correlation indicates that the computed *F*_Slope_ values are able to accurately predict the expected results, proving the efficacy of the Morgenstern-Price method in finite slope stability analysis.

**Fig. 9:**
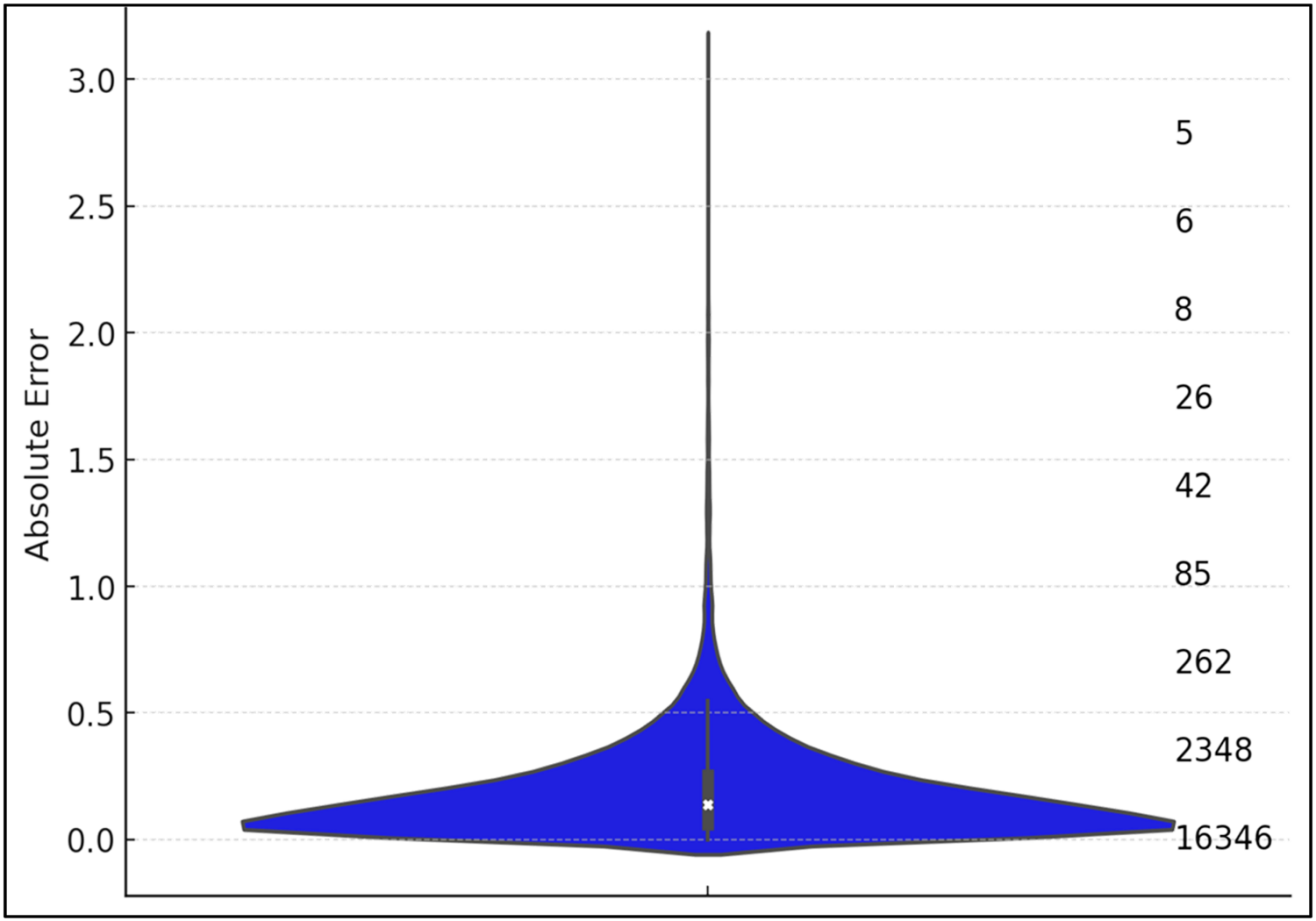
Violin plot illustrating error concentration.

A violin plot was applied for visual representation of the distribution of validation prediction errors. The plot (Fig. 9) shows that the majority of the errors are in the range of 0 to 0.5, reflecting small deviation from the anticipated *F*_Slope_ values. The small spread of errors indicates that the Morgenstern-Price approach is consistently accurate with low variability. The representation of density in the violin plot again supports that severe errors are infrequent, substantiating the consistency and robustness of the deterministic LEM-based results across diverse slope conditions.

R^2^ of 94% guarantees that the Morgenstern-Price procedure gives the *F*_Slope_ with high accuracy and minimal deviation from standard values. The process was run on complete dataset, and the result was consistent and therefore appropriate for various geotechnical applications. This validation approach is consistent with reliability-based model verification frameworks adopted in recent ML–geotechnical studies, where model robustness is demonstrated through independent data validation^30^.

Although only a single published benchmark study^28^ was used for direct external validation of the Morgenstern–Price implementation, this case was selected deliberately because it is independent, incorporates pseudo-static seismic loading, and is well aligned with the modeling assumptions adopted in the present work. Beyond this external comparison, the robustness of the deterministic formulation is supported by extensive internal validation across a broad parametric space, sensitivity patterns that agree with established geotechnical theory, and stable statistical performance observed over the entire synthetic dataset. Nevertheless, it is acknowledged that validation against additional benchmark problems or field-monitored slope case histories would further strengthen the assessment, and such extensions are identified as a natural direction for future research.

### ML results

ML techniques have been commonly applied in geotechnical engineering practice with predictive capability to enhance traditional LEM. Here, we trained and tested various ML models with the aim of predicting *F*_Slope_ for finite slope analysis. The models were compared in terms of performance based on diverse statistical metrics to ascertain accuracy and reliability. Performance evaluation considered several important statistical indicators like R, R^2^, adjusted R^2^, NS, WMAPE, MAE, MBE, RMSE, WI, and LMI (Table 7). These indicators provide a complete picture of the forecasting ability and error range of the models.

**Table 7:** Performance metrics for T_RG_ and T_SG_ datasets of the ML models employed.

Model	Dataset	R	R^2^	RMSE	WMAPE	Adj R^2^	NS	MAE	MBE	WI	LMI
RF	T_RG_	0.999	0.998	0.03	0.02	0.998	0.998	0.016	0	0.999	0.977
	T_SG_	0.993	0.985	0.084	0.053	0.985	0.985	0.042	0	0.996	0.937
AdaBoost	T_RG_	0.977	0.952	0.146	0.145	0.952	0.952	0.114	0.034	0.987	0.887
	T_SG_	0.973	0.944	0.162	0.15	0.944	0.944	0.119	0.034	0.985	0.878
GBoost	T_RG_	1	0.999	0.017	0.014	0.999	0.999	0.011	0	1	0.987
	T_SG_	0.999	0.997	0.037	0.021	0.997	0.997	0.017	0	0.999	0.973
XGBoost	T_RG_	1	0.999	0.021	0.019	0.999	0.999	0.015	0	1	0.984
	T_SG_	0.998	0.997	0.039	0.028	0.997	0.997	0.022	0	0.999	0.971
CatBoost	T_RG_	0.999	0.999	0.005	0.004	0.999	0.999	0.003	0	0.999	0.996
	T_SG_	0.999	0.999	0.014	0.006	0.999	0.999	0.004	0	0.999	0.989
DT	T_RG_	1	1	0	0	1	1	0	0	1	1
	T_SG_	0.98	0.96	0.137	0.086	0.96	0.96	0.068	0	0.99	0.899
XT	T_RG_	1	1	0	0	1	1	0	0	1	1
	T_SG_	0.994	0.988	0.074	0.044	0.988	0.988	0.035	0	0.997	0.945
KNN	T_RG_	0.93	0.849	0.258	0.19	0.849	0.849	0.149	− 0.024	0.952	0.785
	T_SG_	0.883	0.758	0.336	0.235	0.758	0.758	0.186	− 0.033	0.916	0.718
ANN	T_RG_	0.999	0.998	0.026	0.024	0.998	0.998	0.019	− 0.001	1	0.98
	T_SG_	0.999	0.998	0.032	0.025	0.998	0.998	0.02	− 0.002	0.999	0.976

Among the models that were tested, RF performed exceptionally well with R^2^ of 0.985 and RMSE of 0.084. It was extremely accurate with the minimum levels of errors, and thus, it is a good choice for *F*_Slope_ prediction. AdaBoost, although still extremely good, was not as accurate with R^2^ of 0.944 and significantly higher WMAPE of 0.15 with higher variations in levels of errors than other competing models. GBoost and XGBoost performed extremely well with R^2^ of 0.997 and RMSE of 0.037 and 0.039, respectively. Both models were extremely robust with minimum levels of error margins, confirming their performance in slope stability prediction. CatBoost, however, was the best-performing model with R^2^ of 0.999, extremely low RMSE of 0.014, and lowest WMAPE of 0.006. Furthermore, CatBoost also had the lowest MAE of 0.004, indicating its highest level of accuracy in *F*_Slope_ forecasts. Overall, although all ML models were performing extremely high, CatBoost was the best by offering the best combination of prediction accuracy and smallest error. The results suggest that ML techniques, and specifically CatBoost, may be extremely good complements to traditional LEM techniques to significantly improve the efficiency and accuracy of slope stability analysis in geotechnical engineering applications.

To effectively assess and measure the effectiveness of the machine learning models that were used for the purposes of prediction, a comparative analysis was done in depth between the predicted *F*_Slope_ derived from the models and the actual *F*_Slope_. These graphical plots are very vital as they graphically show the degree of accuracy with which the model’s approximations correspond to the actual *F*_Slope_ values. The ideal or best model will be characterized by a cluster of points that closely follow the 45-degree reference line on the graph. This specific alignment indicates that there is a very small difference between the true real values and the predicted values obtained from the model, indicating its high level of predictive accuracy.

In addition to conventional evaluation indicators such as R^2^, RMSE, and MAE, information-theoretic criteria namely the Akaike Information Criterion (AIC) and Optimized Bayesian Criterion (OBC) were calculated to assess model parsimony and robustness^31,32^. These metrics penalize model complexity while rewarding predictive accuracy, thereby providing a balanced comparison among competing algorithms. Table 8 summarizes the computed AIC and OBC values for both training and testing datasets.

**Table 8:** Information-theoretic performance evaluation of ML models. Lower (more negative) AIC and OBC values indicate better model fit and parsimony.

Model	AIC	OBC
Training		
RF	− 98,052.4	− 97,976.93
AdaBoost	− 53,874.48	− 53,799.01
GBM	− 113,801.55	− 113,726.09
XGBoost	− 108,286.63	− 108,211.16
CatBoost	− 149,138.5	− 149,063.04
DT	− 1,019,277.23	− 1,019,239.50
XT	− 961,420.88	− 961,345.41
KNN	− 37,957.002	− 37,934.36
ANN	− 101,974.61	− 101,861.41
Testing		
RF	− 29,663.72	− 29,596.72
AdaBoost	− 21,846.71	− 21,779.72
GBM	− 39,684.66	− 39,617.66
XGBoost	− 38,786.02	− 38,719.03
CatBoost	− 50,846.54	− 50,779.54
DT	− 23,849.24	− 23,815.74
XT	− 31,229.59	− 31,162.6
KNN	− 13,065.35	− 13,045.26
ANN	− 41,251.67	− 41,151.18

The results indicate that the CatBoost model achieved the lowest AIC (− 50,846.54) and OBC (− 50,779.55) on the testing dataset, confirming its superior balance between model complexity and predictive performance. The rankings derived from AIC and OBC are consistent with those obtained from R^2^, RMSE, and MAE, reinforcing the reliability of CatBoost as the best-performing model for slope stability prediction.

**Fig. 10:**
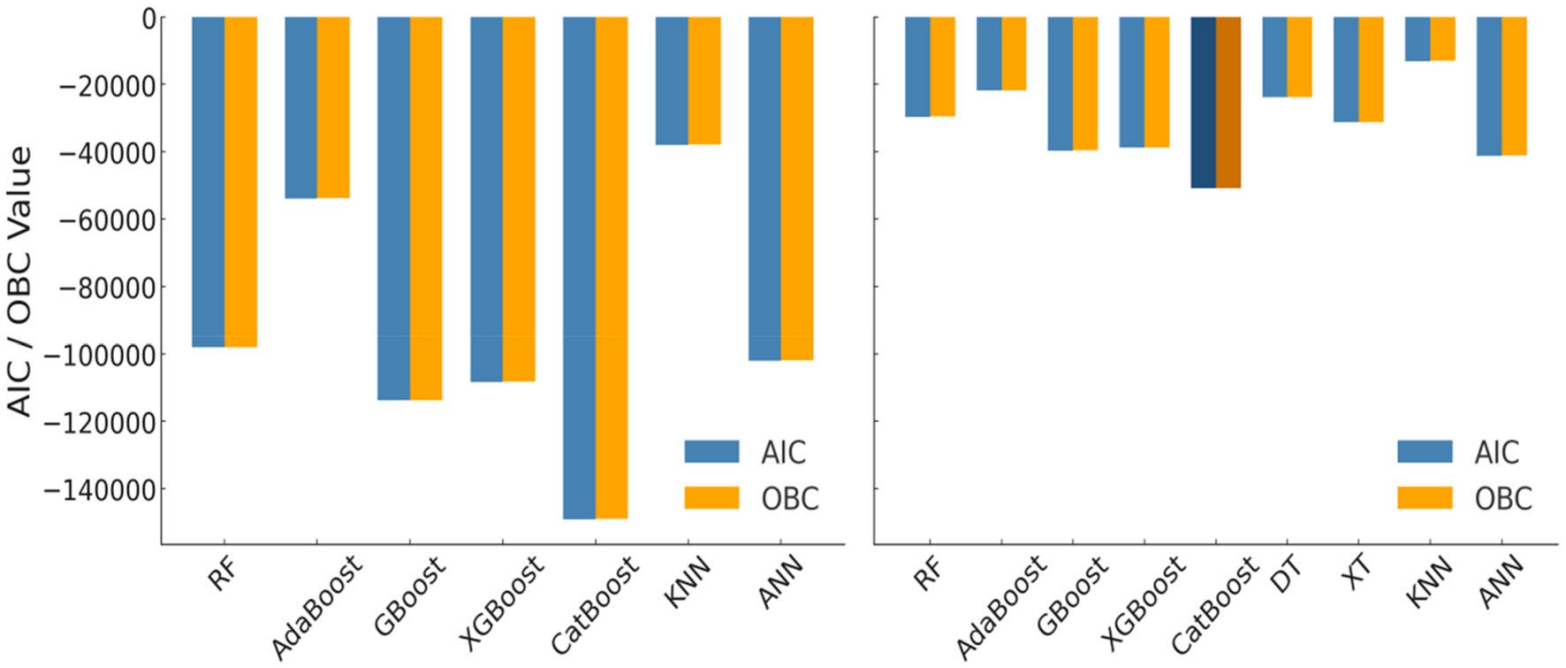
Comparison of AIC and OBC values across machine learning models for training and testing datasets.

A visual comparison of AIC and OBC values across all machine learning models is presented in Fig. 10. The bar chart clearly illustrates that CatBoost, XGBoost, and GBM exhibit the lowest AIC and OBC values among all algorithms, reflecting their high predictive accuracy with minimal complexity penalties. In contrast, DT and XT display extremely large negative AIC and OBC scores during training, that is an indication of overfitting, as these models tend to memorize training data and achieve artificially low residuals. These overfitted values distort the scale of comparison; therefore, DT and XT are excluded from the training dataset plot but retained in the testing dataset for completeness. Overall, the consistency of CatBoost’s superior performance across both datasets and all evaluation metrics underscores its robustness and generalization ability for slope stability prediction.

**Fig. 11:**
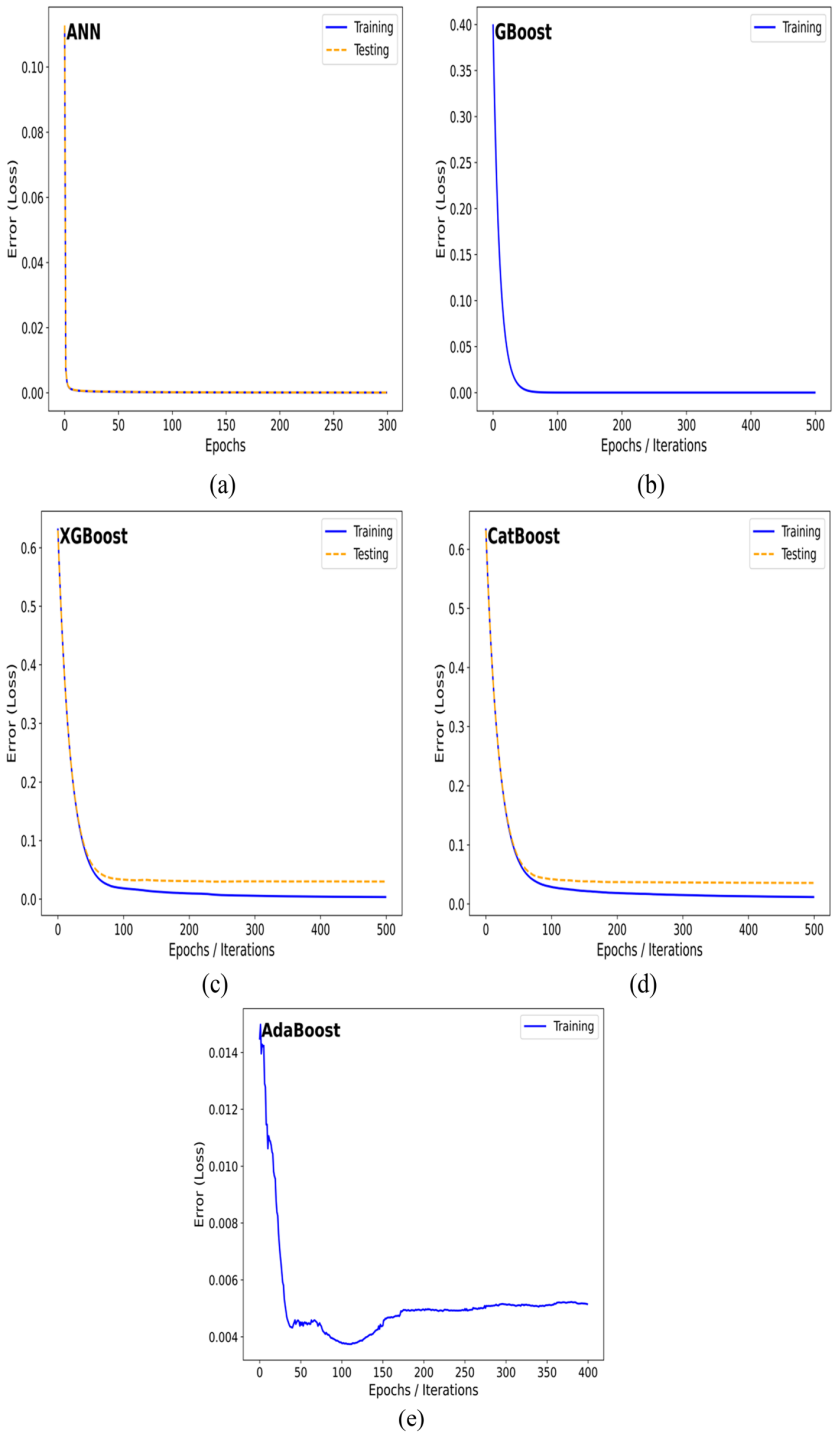
Convergence behavior of the trained machine learning models (**a**) ANN (**b**) GBoost (**c**) XGBoost (**d**) CatBoost (**e**) AdaBoost showing variation of Loss with epochs or iterations.

Furthermore, the convergence behavior of the developed ML models was analyzed to assess training stability, learning efficiency, and generalization performance. Figure 11 presents the convergence curves for the five key algorithms i.e., ANN, AdaBoost, GBoost, XGBoost, and CatBoost, showing the evolution of Mean Squared Error (MSE) over epochs or iterations. Models that exhibited severe overfitting or unstable loss patterns, were excluded from this analysis for clarity.

Overall, all models demonstrated smooth and stable convergence, confirming proper learning without oscillations or divergence. The ANN showed a rapid decline in both training and testing loss during the early epochs, reaching stable minima with minimal gap between curves, indicating efficient generalization. Similarly, XGBoost and CatBoost achieved monotonic decreases in loss with closely aligned training and testing trends, reflecting excellent learning control and regularization. Their early stabilization around 100 iterations highlights their computational efficiency and robustness in modeling nonlinear slope stability behavior.

**Fig. 12:**
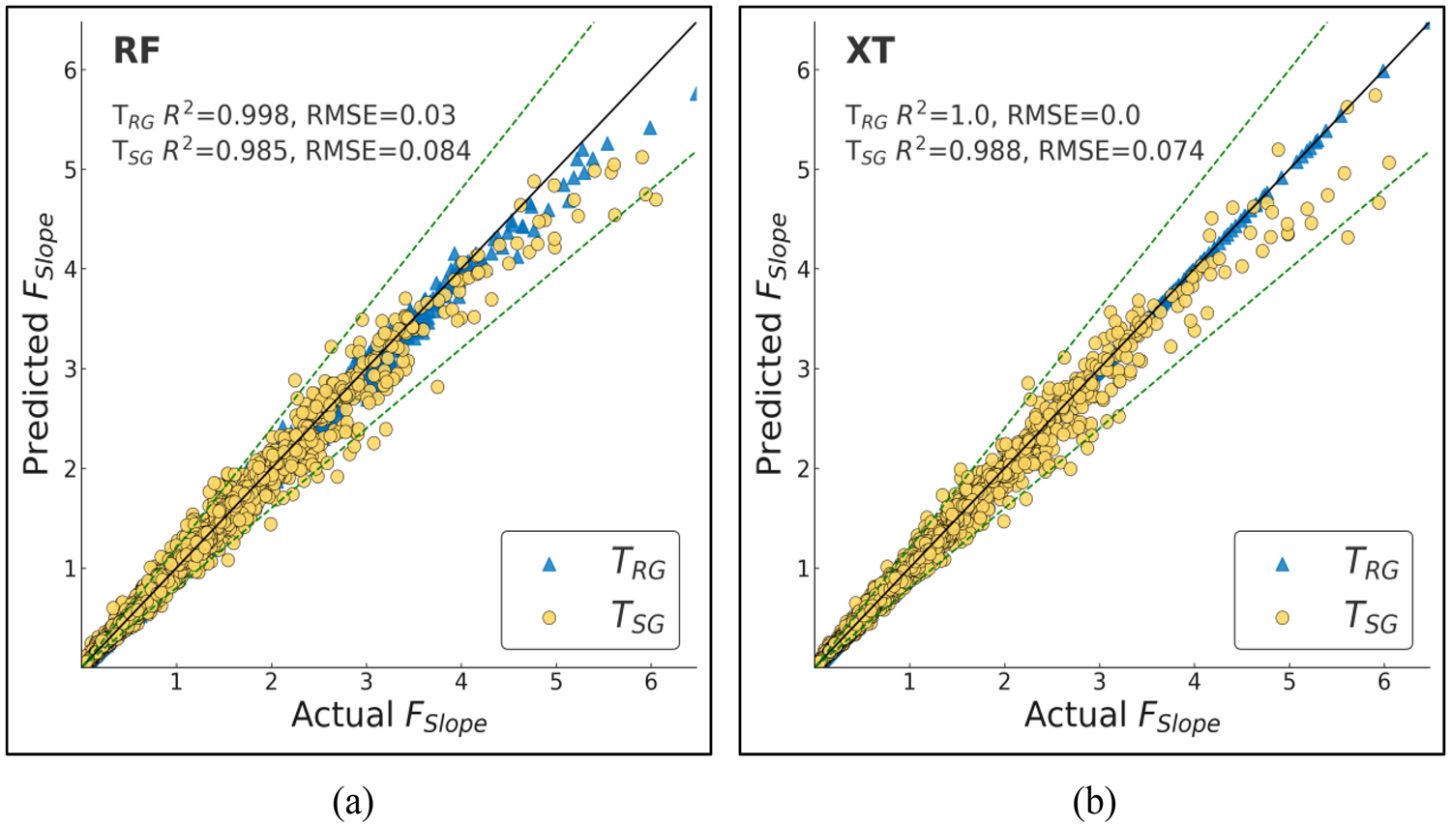
Actual vs predicted scatter plots of bagging models (**a**) RF (**b**) XT.

In contrast, GBoost and AdaBoost exhibited slower and slightly fluctuating convergence patterns due to their iterative additive nature and sensitivity to weak learners. Despite this, both models ultimately stabilized at low loss values, suggesting reliable performance without significant overfitting. The overall comparison indicates that XGBoost and CatBoost not only converged faster but also maintained optimal balance between learning complexity and predictive accuracy, reinforcing their superiority for finite slope stability prediction (Fig. 12).

**Fig. 13:**
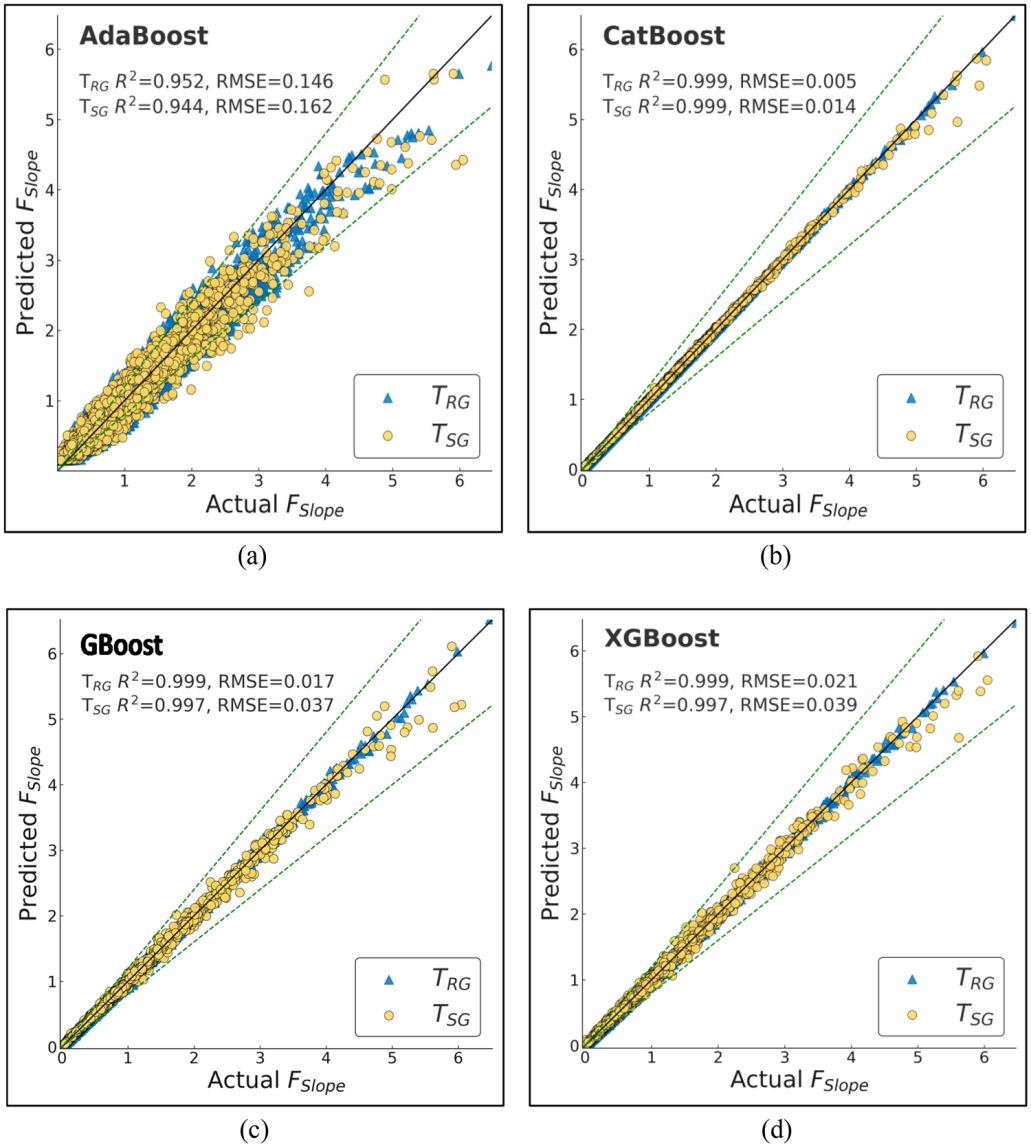
Actual vs predicted scatter plots of boosting models (**a**) AdaBoost (**b**) CatBoost (**c**) GBoost (**d**) XGBoost.

Among all the models, CatBoost made the most precise predictions, as clear from its actual vs. predicted graph, Fig. 13b. The trend of the points closely follows the diagonal reference line, indicating high agreement between the predictions and actual values. This is proof that CatBoost can effectively model the complex relationships in the dataset with very minor generalization errors.

Boosting algorithms, i.e., GBoost and XGBoost (Fig. 13c, d), performed just as well as far as prediction is concerned. Their line plots indicate decent matching of the ideal prediction line, although slightly more scattered than CatBoost’s. It shows that the models are capable of detecting underlying trends but add very little variations at some points. Overall, the boosting algorithms performed well; however, the AdaBoost model (Fig. 13a) showed relatively greater scatter compared to the other boosting-based methods.

**Fig. 14:**
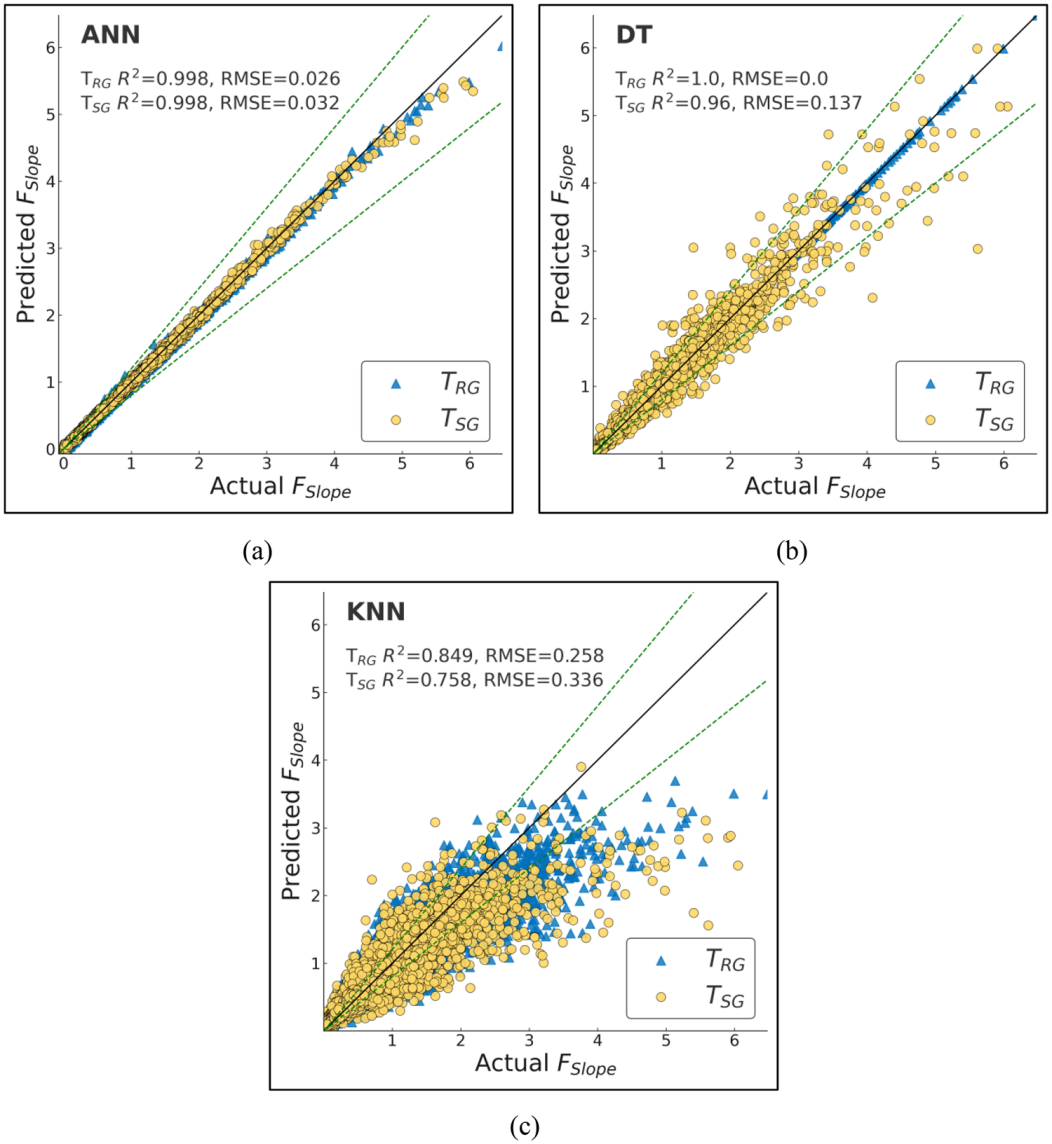
Actual vs predicted scatter plots of benchmark models (**a**) ANN (**b**) DT (**c**) KNN.

Conversely, benchmark models like DT and KNN (Fig. 14) were more scattered in actual vs. predicted. The spread indicates greater error limits and reduced predictability. The models were poor at generalizing and thus generated more variability in their predictions. The benchmarking models showed great accuracy (Fig. 12), particularly the XT, which achieved perfect accuracy on the T_RG_ dataset likely due to overfitting as discussed in previous section and near-perfect accuracy when testing.

Overall, the analysis supports CatBoost as the highest-performing model attributed to the satisfactory fit along the accuracy reference line. The findings of the prediction analyses also validate the need for model selection in the best prediction accuracy, especially in the case of complex data where flexibility and robustness are of most concern.

**Fig. 15:**
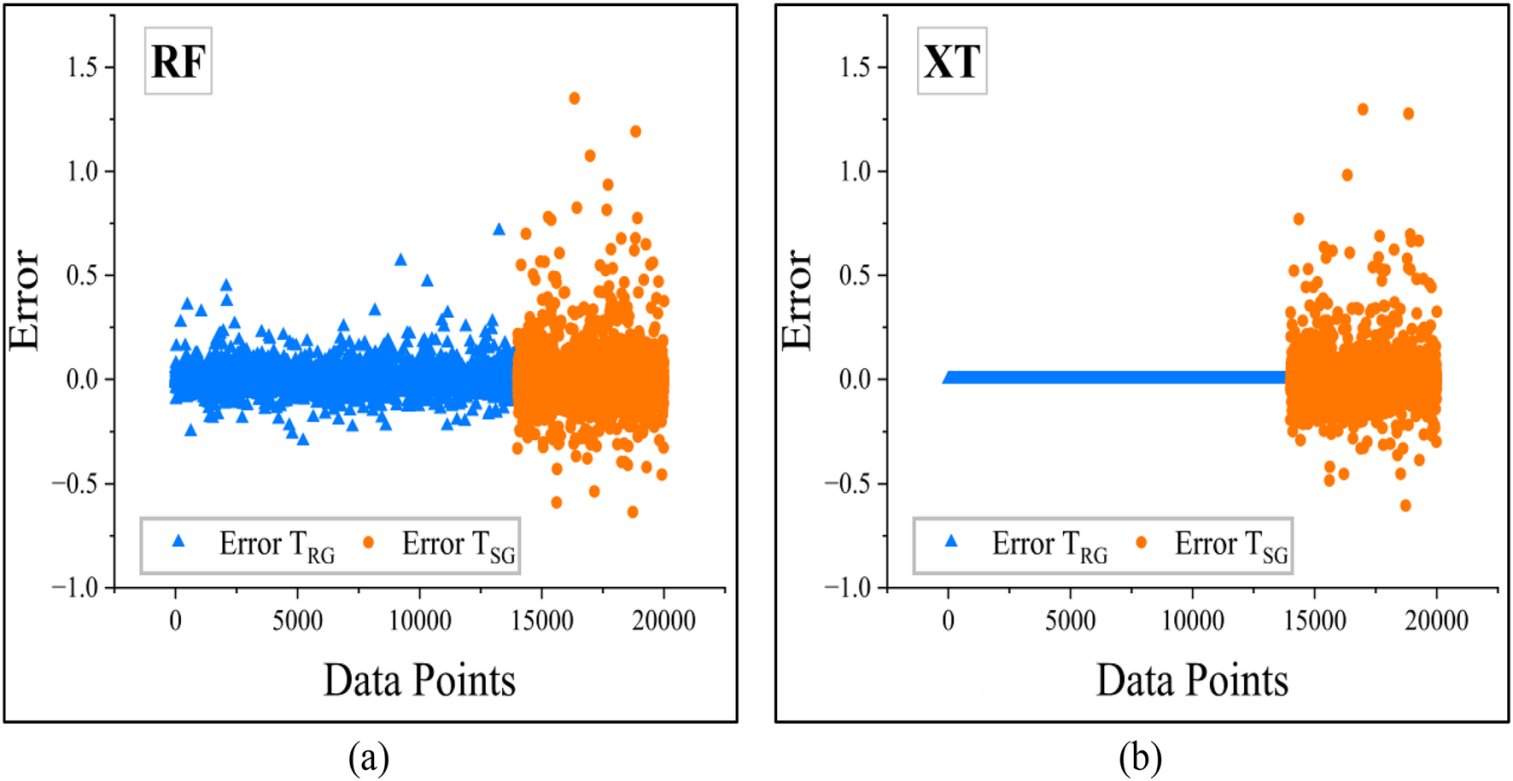
Plots illustrating error for bagging models (**a**) RF (**b**) XT.

In addition, in order to evaluate entirely the accuracy of various machine learning algorithms in predicting slope stability, an error analysis was performed in relation to their predictive accuracy. The difference between the predicted and actual values is a measure of the effectiveness of each algorithm in detecting latent patterns in the dataset (Fig. 15).

**Fig. 16:**
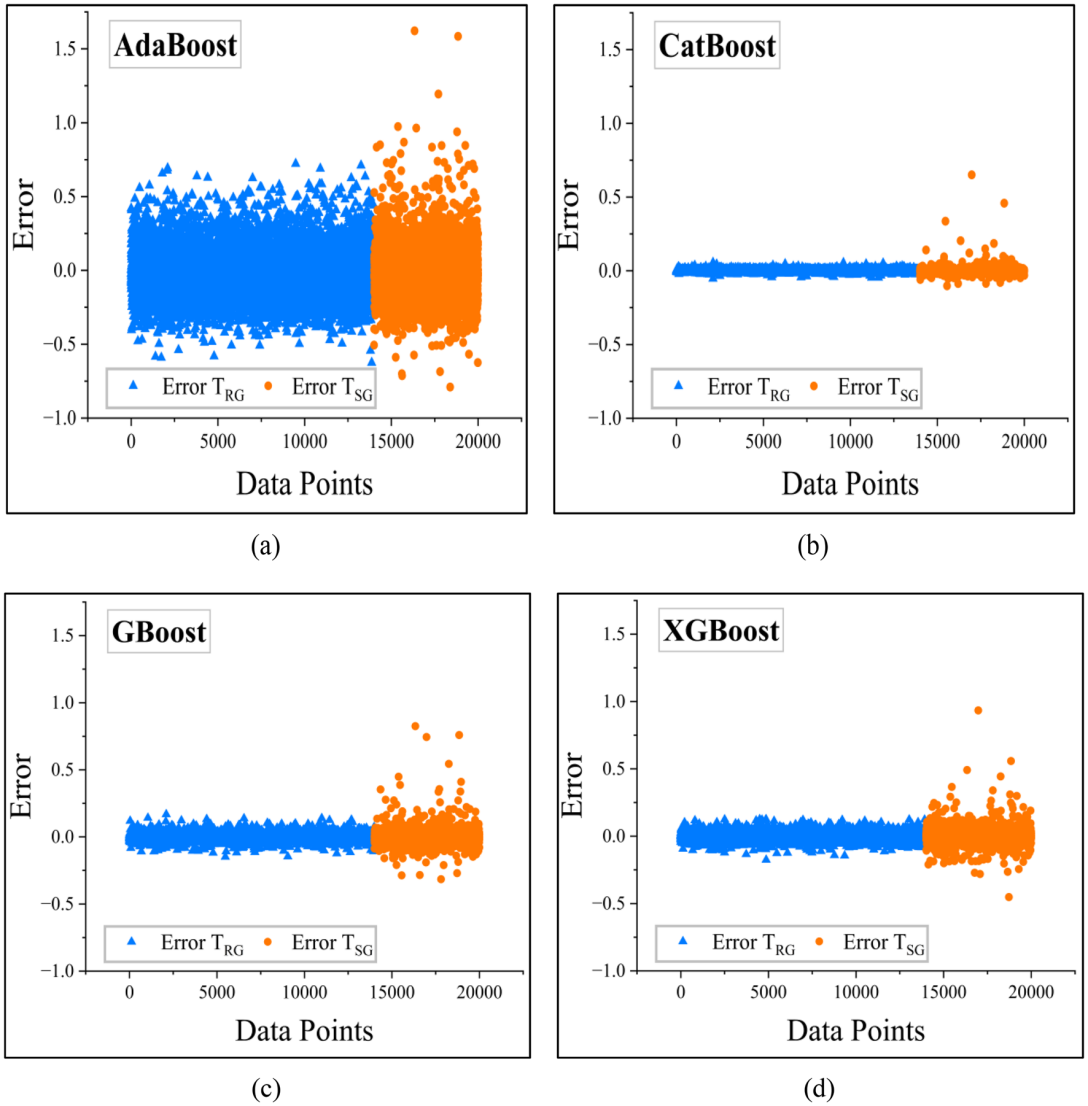
Plots illustrating error for boosting models (**a**) AdaBoost (**b**) CatBoost (**c**) GBoost (**d**) XGBoost.

Out of all the models, CatBoost possessed the smallest error margins, as it can be observed from Fig. 16b. This reflects high precision and generalizability, and hence the best model for finite slope stability analysis.

The rest of the boosting models, such as AdaBoost, XGBoost, and GBoost, also performed well in terms of error prediction. The error distribution of AdaBoost is presented in Fig. 16a, where the residual error dispersion was relatively larger. However, the error values of XGBoost and GBoost, as presented in Fig. 16c,d, respectively, were smaller. Although their predicted values had a slightly larger variance than CatBoost, they were still very close to the true *F*_Slope_ values, indicating their high adaptability to the data.

**Fig. 17:**
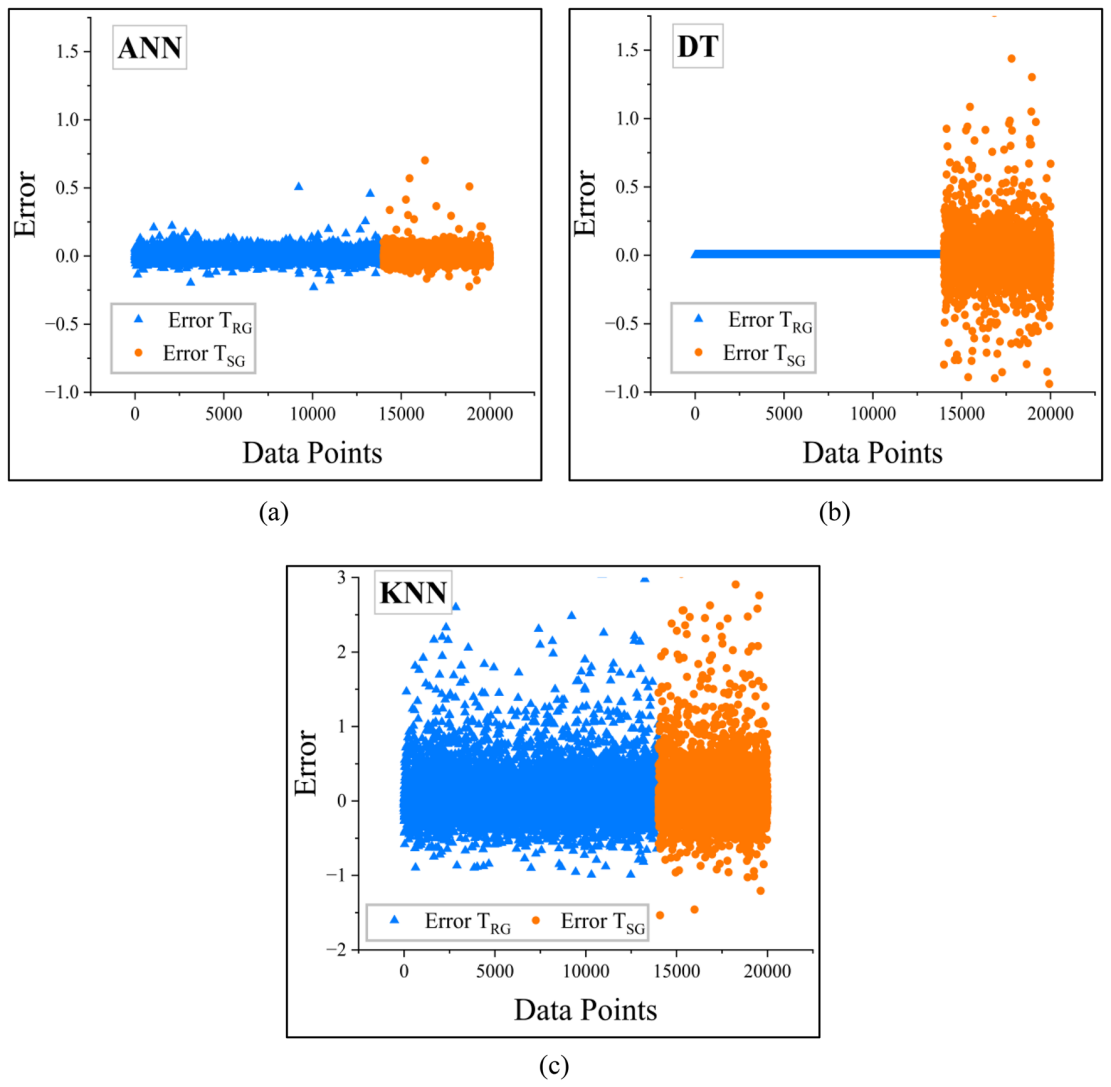
Plots illustrating error for benchmark models (**a**) ANN (**b**) DT (**c**) KNN.

Although the bagging models performed well in T_RG_, they performed poorly in the T_SG_ as can be seen from Fig. 15 compared to benchmark ANN- Fig. 17a. However, other benchmarking models DT and KNN models had greater deviations, as can be seen from Fig. 17b,c respectively. The greater scatter indicates variability of predictions, that is, these models tend to overfit or underfit slope stability data.

The error analysis confirms that ensemble-based models, particularly boosting algorithms, have stronger prediction performance than other machine learning algorithms. The findings suggest the necessity of choosing the models with a good generalization capability so that the predictions can be stable and accurate for geotechnical applications.

### Automated framework performance

The automated framework was developed using geotechnically realistic parameter ranges synthesized from the literature, with random sampling employed to ensure broad coverage of slope geometries, material properties, and seismic conditions. To preserve both computational rigor and statistical representativeness, the entire automated pipeline from data generation and MP-LEM computation to machine-learning training, testing, and optimal model selection was executed on the complete 100,000-case synthetic dataset. Implementation details and reproducibility considerations of the Python-based automated system are provided in the ‘Supplementary Information’.

**Fig. 18:**
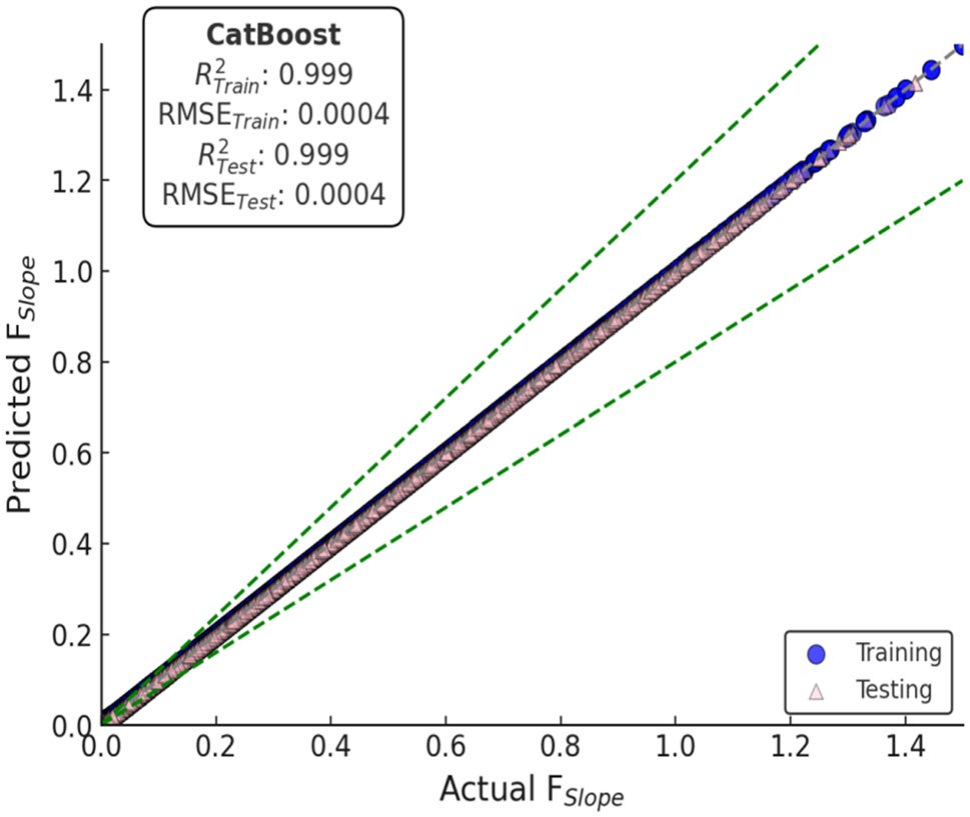
Actual vs predicted of automated model (CatBoost).

Within the automated framework, multiple machine-learning algorithms were trained and evaluated for *F*_Slope_ prediction. Among them, CatBoost consistently emerged as the best-performing model, exhibiting excellent accuracy and numerical stability across both training (T_RG_) and testing (T_SG_) phases. The predicted versus actual *F*_Slope_ plot for the CatBoost model (Fig. 18) shows a tight clustering of data points along the 1:1 reference line, indicating minimal deviation between predicted values and MP-LEM-computed *F*_Slope_ and confirming the model’s strong ability to replicate deterministic slope-stability solutions.

**Fig. 19:**
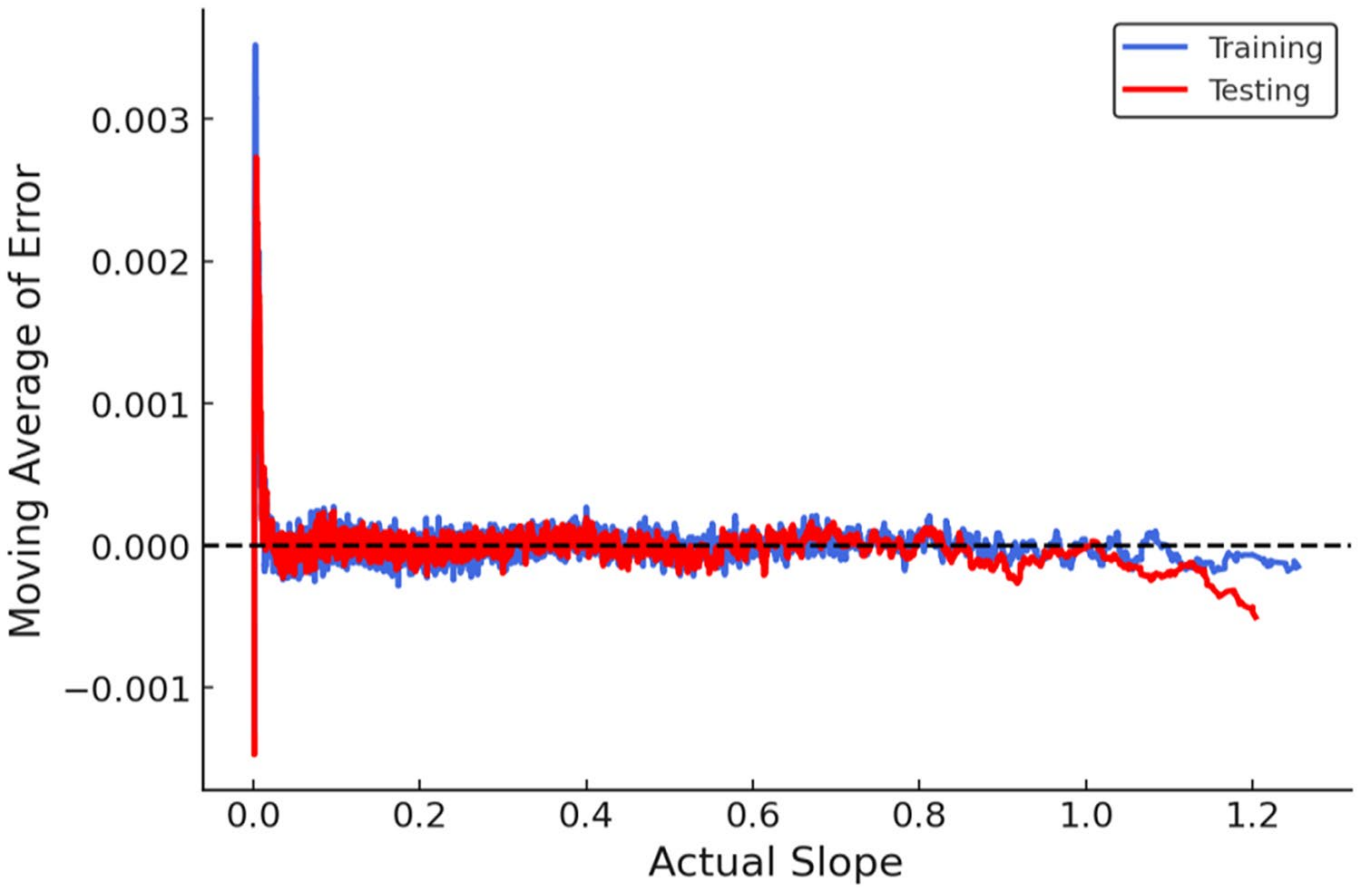
Moving average error plot illustrating error of automated Model (CatBoost) against actual *F*_Slope_.

The moving-average error plot (Fig. 19) further demonstrates the robustness of the automated CatBoost model. The zero-error reference line represents ideal predictions, and both T_RG_ and T_SG_ curves remain closely aligned with this reference across the *F*_Slope_ range. Despite the large-scale automated analysis, error magnitudes remain extremely low (RMSE = 0.0004, MAE = 0.0002), with negligible mean bias error, indicating the absence of systematic prediction bias and confirming high numerical reliability.

**Fig. 20:**
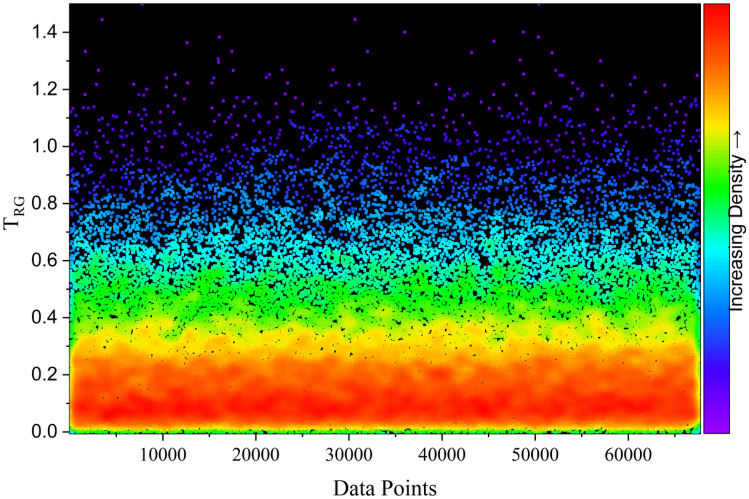
Density dots illustrating the predicted *F*_Slope_ distribution of T_RG_ dataset.

**Fig. 21:**
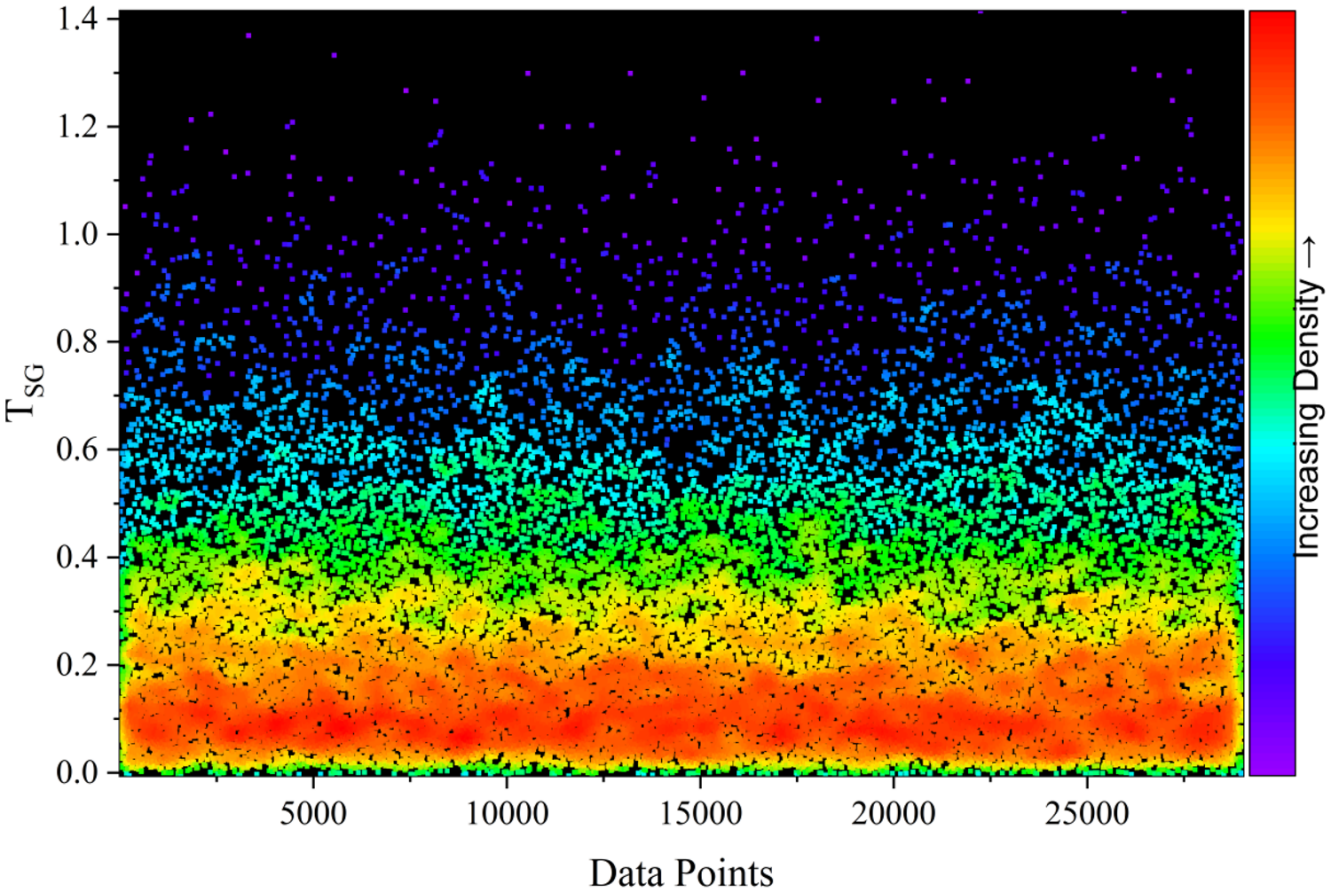
Density dots illustrating the predicted *F*_Slope_ distribution of T_SG_ dataset.

To visualize the global distribution of *F*_Slope_ across the full parameter space, density-based scatter plots were generated using the complete 100,000-case dataset (Figs. 20, 21). These plots serve a descriptive purpose, illustrating the underlying distribution of slope stability conditions captured by the automated framework. In both T_RG_ and T_SG_ datasets, *F*_Slope_ values are densely concentrated in the lower range (approximately 0–0.3), indicating a predominance of unstable slope conditions, while higher *F*_Slope_ values are progressively less dense, reflecting fewer highly stable cases.

The similarity in density patterns between T_RG_ and T_SG_ datasets confirms the representativeness of the automated data split. However, the observed concentration toward lower *F*_Slope_ values also highlights an inherent imbalance in the dataset, with relatively fewer stable slope instances. While this bias enhances predictive precision in high-risk failure zones, it may limit generalization in very stable conditions. Future extensions of the framework may address this limitation through targeted data augmentation or stratified resampling strategies.

Overall, the proposed automated modeling strategy significantly enhances computational efficiency while maintaining high predictive accuracy for finite slope stability assessment. By integrating a physics-based modified Morgenstern–Price formulation with advanced machine-learning algorithms in a fully automated and reproducible Python workflow, the framework provides a scalable and flexible alternative to traditional deterministic approaches. This hybrid methodology enables rapid assessment across diverse geotechnical and seismic conditions and establishes a strong foundation for future extensions incorporating probabilistic analysis, real-world case histories, and three-dimensional effects.

### Overfitting, data imbalance, and model generalization

The high predictive performance achieved by several machine-learning models, particularly ensemble-based learners, warrants careful interpretation. Although near-perfect performance metrics may raise concerns regarding potential overfitting, it is important to recognize that *F*_Slope_ is a deterministic output computed from a physics-based limit equilibrium formulation. Consequently, the ML models are effectively learning a smooth functional relationship governed by the underlying mechanics of slope stability, rather than fitting noisy observational data, which naturally leads to very high predictive accuracy when predictions are made within the sampled parameter space. To prevent data leakage during model evaluation, normalization parameters were derived exclusively from the training dataset and subsequently applied to the testing dataset, ensuring unbiased performance assessment.

Nevertheless, evidence of model-specific overfitting behavior is observed. In particular, the DT and XT models exhibit near-perfect performance during training but show comparatively larger error dispersion and reduced stability during testing. This behavior is consistent with the tendency of tree-based models without sufficient regularization to memorize training patterns, especially when trained on large synthetic datasets derived from deterministic formulations. By contrast, CatBoost, along with other gradient-boosting models, demonstrates consistently high performance across both training and testing datasets with minimal degradation, indicating superior generalization capability. This robustness is attributed to its ordered boosting strategy, built-in regularization, and resistance to overfitting, which is further supported by tighter clustering around the 1:1 line in the scatter plots.

In addition to model structure, data imbalance plays an important role in generalization behavior. The *F*_Slope_ distribution is strongly skewed toward lower values, with unstable and marginally stable slope conditions being more densely represented than highly stable cases. Although stratified sampling was employed to ensure representative training and testing subsets, the sparse presence of high *F*_Slope_ values may reduce predictive reliability in these regions, as reflected by increased uncertainty in density-based visualizations. Furthermore, the proposed ML models are primarily intended for interpolation within the defined parameter ranges rather than extrapolation beyond them. Predictions for slope configurations outside the trained input space such as extreme geometries, atypical soil properties, or complex hydro-mechanical conditions should therefore be treated with caution. Future work may address these limitations through targeted data augmentation, balanced resampling strategies, and validation against real case histories to further enhance model robustness and generalization.

## Summary and conclusion

This study presents a fully automated hybrid framework that integrates a simplified Morgenstern–Price limit equilibrium formulation with machine-learning techniques for rapid and reliable finite slope stability assessment under static and seismic conditions. A large synthetic dataset spanning geotechnically realistic soil, geometric, hydraulic, and seismic parameter ranges was generated, and corresponding *F*_Slope_ values were computed using the Morgenstern–Price method. Sensitivity analysis identified slope angle (β), slope height (*H*), and cohesion (c′) as the dominant parameters governing slope stability, in strong agreement with established principles of soil mechanics and slope engineering.

To reduce the computational burden associated with repeated limit equilibrium calculations, multiple machine-learning algorithms were evaluated as surrogate models for *F*_Slope_ prediction. Among the tested models, CatBoost demonstrated the most robust and consistent performance, achieving high accuracy across both training and testing datasets with negligible bias and superior generalization behavior. Comparative analysis revealed that simpler tree-based models, such as Decision Tree and Extra Trees, were more susceptible to overfitting, whereas CatBoost maintained stable predictive performance due to its ordered boosting strategy and built-in regularization. Independent verification of the deterministic Morgenstern–Price implementation against published benchmark results yielded an R^2^ value of 0.94, confirming the reliability of the underlying physics-based formulation on which the surrogate models were trained.

The complete workflow was implemented as a scalable and reproducible Python-based automated pipeline, enabling efficient data generation, preprocessing, model training, and model selection. It is emphasized that the machine-learning models developed in this study are trained to emulate the Morgenstern–Price limit equilibrium solver and are therefore validated as computational surrogates of this deterministic formulation, rather than as direct predictors of field-measured slope performance. Within this scope, the proposed framework offers a computationally efficient alternative to conventional limit equilibrium analysis for large-scale parametric studies, preliminary design assessment, and decision support. While the surrogate models are primarily intended for interpolation within the trained parameter space, future work may extend the framework to validation against real case histories, independent numerical models, probabilistic analyses, and three-dimensional slope stability problems to further enhance its applicability in practical geotechnical engineering.

## Supplementary Information

Supplementary Information.

## Data Availability

The dataset employed in this research is synthetically created and can be completely replicated by using the Python-based automated workflow developed by the authors. The entire workflow employed in this research, from data generation to evaluation, will be made publicly available upon acceptance of the manuscript. Until then, the data and code can be obtained from the corresponding author upon reasonable request.

## List of symbols

*F*_Slope_: Factor of safety
LEM: Limit equilibrium method
FEM: Finite element method
MP: Morgenstern-Price
ML: Machine learning
T_RG_: Training dataset
T_SG_: Testing dataset
ANN: Artificial neural network
RF: Random forest
DT: Decision tree
KNN: K-nearest neighbor
XT: Extra trees
XGBoost: EXtreme gradient boosting
GBoost: Gradient boosting
AdaBoost: Adaptive boosting
CatBoost: Categorical boosting
γ: Unit weight of soil
H: Slope height
c′: Cohesion
ϕ′: Internal friction angle
β: Slope angle
k_h_: Horizontal seismic coefficient
k_v_: Vertical seismic coefficient
μ: Pore pressure ratio
λ: Interslice force scaling factor
R: Pearson correlation coefficient
R^2^: Coefficient of determination
Adj. R^2^: Adjusted coefficient of determination
RMSE: Root mean squared error
MAE: Mean absolute error
MBE: Mean bias error
WMAPE: Weighted mean absolute percentage error
WI: Willmott’s index of agreement
NS: Nash–Sutcliffe efficiency coefficient
LMI: Legates and McCabe’s index
