# Supplementary material for: Automated slope stability assessment using modified Morgenstern-Price method and machine learning integration
Source: Sci Rep. 2026 Feb 19;16:9952. doi: 10.1038/s41598-026-38670-w (PMC13022241; doi:10.1038/s41598-026-38670-w)
Supplement: Supplementary file 1 — Supplementary Information. [file 41598_2026_38670_MOESM1_ESM.pdf]

SUPPLEMENTARY INFORMATION:

REPRODUCIBILITY AND IMPLEMENTATION DETAILS OF THE PYTHON-BASED SURROGATE FRAMEWORK

This Supplementary Infromation outlines an automated modeling approach made for the purpose of predicting F_{Slope} in finite slope stability analysis. The implementation of geotechnical parameters for the provided Python code is structured, data is normalized, ML predictions are made, and the performances of the employed ML algorithms are subsequently evaluated. Following is the code explained step by step:

Step 1: *Import Libraries Needed*

The code starts with importing the needed libraries for handling data, machine learning, and performance measurement.

```
import numpy as np
import itertools
from tqdm import tqdm
from sklearn.model_selection import train_test_split
from sklearn.preprocessing import MinMaxScaler
from sklearn.ensemble import (
    RandomForestRegressor,
    ExtraTreesRegressor,
    GradientBoostingRegressor,
    AdaBoostRegressor
)
from sklearn.tree import DecisionTreeRegressor
from sklearn.neighbors import KNeighborsRegressor
from sklearn.metrics import r2_score, mean_squared_error, mean_absolute_error
import xgboost as xgb
import catboost as cb
import tensorflow as tf
from tensorflow import keras
```

Figure 1A: Importing of necessary libraries

Step 2: *Define the F_{Slope} Calculation Function*

The *calculate_fos_morgenstern_price* function computes the F_{Slope} using the Morgenstern-Price method, which is a popular slope stability analysis technique.

```python
# Function to calculate FSlope using simplified MP-LEM
# -------------------------------------------------
def calculate_fslope_morgenstern_price(c, phi, gamma, H, beta, kh, kv, ru, lambda_):
    dx = 1.0
    beta_rad = np.radians(beta)

    W = gamma * H * dx
    N = W * np.cos(beta_rad) - kh * W * np.sin(beta_rad)
    Ru = ru * gamma * H

    S = np.maximum(c + (N - Ru) * np.tan(np.radians(phi)), 0.001)
    R = (W * np.sin(beta_rad)) + (kv * W)

    FSlope = np.where(R > 0, S / R, 0.001)
    return np.maximum(FSlope, 0.001)
```

Figure 1B: F_{Slope} calculation function

Step 3: *Set Parameter Ranges*

The script assigns parameter ranges for different seismic and soil properties. These parameters have an impact on slope stability and are diversified across a defined range to provide unique T_{RG} data to machine learning.

```python
# Define parameter ranges
# -------------------------------------------------
param_ranges = {
    "Cohesion (c)": np.arange(10, 70, 10),
    "Friction Angle (ϕ)": np.arange(0, 35, 5),
    "Unit Weight (γ)": np.arange(16, 24, 2),
    "Slope Height (H)": np.arange(15, 75, 10),
    "Slope Angle (β)": np.arange(30, 70, 10),
    "Seismic Coefficient (k_h)": [0, 0.2],
    "Seismic Coefficient (k_v)": [0, 0.1],
    "Pore Pressure Ratio (r_u)": [0, 0.2, 0.4],
    "Interslice Force Scaling Factor (λ)": [0, 0.1],
}
```

Figure 1C*:* Parameter ranges

Step 4: *Create Parameter Combinations*

With NumPy's meshgrid, the script creates all combinations of parameter values. These combinations constitute the dataset so that the model learns under a variety of conditions.

```python
# Generate parameter combinations
# -------------------------------------------------
param_values = [param_ranges[key] for key in param_ranges.keys()]
param_combinations = np.array(
    np.meshgrid(*param_values)
).T.reshape(-1, len(param_values))
```

Figure 1D*:* Parameter combination generating function

Step 5: *Calculate F_{Slope} for Every Combination*

The script transforms parameter combinations into a Pandas DataFrame. Additionally, invokes the *calculate_fos_morgenstern_price* function to calculate F_{Slope}. Eventually, stores the calculated values in a new column.

```
# Compute FSlope using MP method
# ------------------------------------------------
df = pd.DataFrame(param_combinations, columns=param_ranges.keys())
df["FSlope_MP_Calculated"] = calculate_fslope_morgenstern_price(*df.T.values)
```

Figure 1E*:* Calculation of F_{Slope}

Step 6: *Normalize Features*

For better model performance, the features in the input are normalized from 0 to 1 with MinMaxScaler.

```
# Normalize input features (fit on training later)
# ------------------------------------------------
X = df.drop(columns=["FSlope_MP_Calculated"])
y = df["FSlope_MP_Calculated"]

scaler = MinMaxScaler()
X_scaled = scaler.fit_transform(X)

df_normalized = pd.DataFrame(X_scaled, columns=X.columns)
df_normalized["FSlope_MP_Calculated"] = y.values
```

Figure 1F*:* Input feature normalization

Step 7: *Divide Data into T_{RG} and T_{SG} Sets*

The data is divided into 70% T_{RG} and 30% T_{SG} to ensure that the models generalize well to unseen data.

```
# Train-test split (70-30)
# ------------------------------------------------
X_train, X_test, y_train, y_test = train_test_split(
    df_normalized.drop(columns=["FSlope_MP_Calculated"]),
    df_normalized["FSlope_MP_Calculated"],
    test_size=0.30,
    random_state=42
)

df_train = pd.concat([X_train, y_train], axis=1)
df_test = pd.concat([X_test, y_test], axis=1)
```

Figure 1G*:* Data split

Step 8: *Train Machine Learning Models*

The script trains and initializes multiple ML models, such as: Random Forest, Extra Trees, Decision Tree, Gradient Boosting, XGBoost, CatBoost, AdaBoost, and KNN. Predictions for T_{RG} and T_{SG} sets are also saved.

```
# Train ML models
# --------------------------------------------------
models = {
    "Random Forest": RandomForestRegressor(),
    "Extra Trees": ExtraTreesRegressor(),
    "Decision Tree": DecisionTreeRegressor(),
    "Gradient Boosting": GradientBoostingRegressor(),
    "XGBoost": xgb.XGBRegressor(),
    "CatBoost": cb.CatBoostRegressor(verbose=0),
    "AdaBoost": AdaBoostRegressor(),
    "KNN": KNeighborsRegressor(),
}

performance_results = []
train_predictions = {"Actual": y_train.values}
test_predictions = {"Actual": y_test.values}

for name, model in models.items():
    model.fit(X_train, y_train)

    y_train_pred = model.predict(X_train)
    y_test_pred = model.predict(X_test)

    train_predictions[name] = y_train_pred
    test_predictions[name] = y_test_pred

    train_metrics = compute_metrics(y_train, y_train_pred, len(y_train), X_train.shape[1])
    test_metrics = compute_metrics(y_test, y_test_pred, len(y_test), X_test.shape[1])

    performance_results.append([name] + train_metrics + test_metrics)
```

Figure 1H*:* ML model training

Step 9: *Calculate Performance Metrics*

The script measures each model's performance by various metrics: R^2, Adjusted R^2, RMSE, MAE, MBE, WI, LMI, WMAPE, NS. Test R^2 and Test RMSE are used to select the best model.

```
# Store performance results
# --------------------------------------------------
df_performance = pd.DataFrame(
    performance_results,
    columns=[
        "Model",
        "Train r", "Train R²", "Train Adj R²", "Train NS", "Train WMAPE",
        "Train MBE", "Train MAE", "Train RMSE", "Train WI", "Train LMI",
        "Test r", "Test R²", "Test Adj R²", "Test NS", "Test WMAPE",
        "Test MBE", "Test MAE", "Test RMSE", "Test WI", "Test LMI",
    ]
)
```

Figure 1I*:* Calculation of performance metrics and best model selection

Step 10: *Best Model Selection and Save Results to Excel*

The results are lastly saved into an Excel file with individual sheets.

```python
# Select best model
# --------------------------------------------------
best_model = df_performance.sort_values(
    by=["Test R²", "Test RMSE"], ascending=[False, True]
).iloc[0]


# --------------------------------------------------
# Save outputs to Excel
# --------------------------------------------------
output_file = "Final_FSlope_ML_Complete.xlsx"
```

Figure 1J*:* Result storage
